# A novel AR translational regulator lncRNA LBCS inhibits castration resistance of prostate cancer

**DOI:** 10.1186/s12943-019-1037-8

**Published:** 2019-06-20

**Authors:** Peng Gu, Xu Chen, Ruihui Xie, Weibin Xie, Li Huang, Wen Dong, Jinli Han, Xiaodong Liu, Jihong Shen, Jian Huang, Tianxin Lin

**Affiliations:** 1 Department of Urology, Sun Yat-sen Memorial Hospital, Sun Yat-sen University, 107. W. Yanjiang Road, Guangzhou, 510120 China; 2 Department of Urology, The 1st Affiliated Hospital of Kunming Medical University, Kunming, 650032 China; 3 Guangdong Provincial Key Laboratory of Malignant Tumor Epigenetics and Gene Regulation, Sun Yat-Sen Memorial Hospital, Sun Yat-Sen University, Guangzhou, 510120 China; 4 RNA Biomedical Institute, Sun Yat-Sen Memorial Hospital, Sun Yat-Sen University, Guangzhou, 510120 China

**Keywords:** Castration resistance prostate Cancer (CRPC), lncRNA LBCS, Castration resistance, Androgen receptor (AR), hnRNPK

## Abstract

**Background:**

Progression to a castration resistance state is the main cause of deaths in prostate cancer (PCa) patients. Androgen Receptor (AR) signaling plays the central role in progression of Castration Resistant Prostate Cancer (CRPC), therefore understanding the mechanisms of AR activation in the milieu of low androgen is critical to discover novel approach to treat CRPC.

**Methods:**

Firstly, we explore the CRPC associated lncRNAs by transcriptome microarray. The expression and clinical features of lnc-LBCS are analyzed in three independent large-scale cohorts. The functional role and mechanism of lnc-LBCS are further investigated by gain and loss of function assays in vitro.

**Results:**

The expression of Lnc-LBCS was lower in CRPC cells lines and tissues. LBCS downregulation was correlated with higher Gleason Score, T stage and poor prognosis of PCa patients. LBCS overexpression decreases, whereas LBCS knockdown increases, the traits of castration resistance in prostate cancer cells under androgen ablated or AR blocked condition. Moreover, knockdown of LBCS was sufficient to activate AR signaling in the absence of androgen by elevating the translation of AR protein. Mechanistically, LBCS interacted directly with hnRNPK to suppress AR translation efficiency by forming complex with hnRNPK and AR mRNA.

**Conclusions:**

Lnc-LBCS functions as a novel AR translational regulator that suppresses castration resistance of prostate cancer by interacting with hnRNPK. This sheds a new insight into the regulation of CRPC by lncRNA mediated AR activation and LBCS-hnRNPK-AR axis provides a promising approach to the treatment of CRPC.

**Electronic supplementary material:**

The online version of this article (10.1186/s12943-019-1037-8) contains supplementary material, which is available to authorized users.

## Introduction

Prostate cancer (PCa) is the most commonly diagnosed malignancy and the second leading cause of male cancer-related death in the United States [1]. Androgen deprivation therapy (ADT) is the first line treatment for patients with metastatic PCa. Despite the high initial response rates, remissions following ADT are temporary as the occurrence of castration-resistant prostate cancer (CRPC) [2]. Accumulating evidence suggests that abnormally activated Androgen Receptor (AR) signaling are involved in nearly all CRPC cases [3]. For instance, the overexpression of AR is found in approximately 80~90% CRPC patients [4], which is thought to preserve sufficient proliferation signaling even under androgen of castration level [4]. However, the mechanism underlying deregulation of AR remains largely unknown. Identifying new molecular mechanisms of aberrant AR activation holds great promise to improve the treatment of CRPC.

Long noncoding RNAs (lncRNAs) are RNA transcripts that are longer than 200 nucleotides but have rare protein coding potential [5]. Emerging evidence has revealed that lncRNAs play key roles in physiological and pathological process, including embryonic development, organ formation, and human disease [5, 6]. Aberrant expression of lncRNAs has been observed in many types of cancers and are believed to play critical roles in regulating proliferation, metastasis and progression of cancer cells [7, 8]. Transcriptome sequencing across PCa cohorts has identified hundreds of lncRNAs that stratify benign, localized, and metastatic PCa samples [9]. These cancer-associated lncRNAs, like protein-coding genes, may serve as bio-markers of the disease and may be involved in prostate cancer progression [9]. Our previous study reveals that lncRNA HOXD-AS1 is overexpressed in CRPC cell lines and promotes CRPC transition by binding with WDR5/MLL1 complex [10]. In particular, lncRNA *PRNCR1* and *PCGEM1* are overexpressed in aggressive PCa and bind successively to the AR to enhance the AR-mediated gene activation program and induce PCa growth [11]. Although the lncRNAs as regulators in PCa are well studied, the role of lncRNA in the regulation of AR protein is still poorly characterized.

In the current study, we discovered that lnc-LBCS is significantly downregulated in CRPC cell lines and tissues of CRPC patients. Furthermore, LBCS inhibits prostate cancer viability under castration condition by repressing AR signaling. Interestingly, LBCS act as the scaffold to recruit hnRNPK to AR mRNA and then inhibit the translation of AR protein. Our findings suggest strongly that LBCS-hnRNPK-AR axis participates in the progression of PCa and is a promising therapeutic target.

## Material and methods

### Cell culture

The cell lines used in this study included the human prostate cancer cells LNCaP (ATCC, Manassas, VA, USA), and the CRPC-like cell LNCaP-Bic and LNCaP-AI. LNCaP cells were cultured in Roswell Park Memorial Institute (RPMI)-1640 (Gibco, Shanghai, China), supplemented with 10% FBS (fetal bovine serum, Shanghai ExCell Biology, China), LNCaP-AI cells were cultured in phenol red free RPMI-1640 containing 10% charcoal stripped FBS (Gibco, Shanghai, China); whereas LNCaP-Bic were cultured with 20 μM bicalutamide (Sigma, St. Louis, MI, USA). All media were supplemented with 1% penicillin/streptomycin. LNCaP cells were cultured in phenol red free medium with charcoal striped FBS for at least 2 days before any experiment. Cells were grown in a humidified atmosphere of 5% CO_2_ at 37 °C. The construction of two CRPC LNCaP sublines was described in our previous article [10].

### Human tissue samples

A total of 130 cases paraffin embedded PCa tissues and 32 cases of BPH tissues, termed Cohort 1, were obtained by surgery or needle biopsy with the written consent of patients who underwent surgery at Sun Yat-sen Memorial Hospital, Sun Yat-sen University. All the samples were pathologically confirmed as prostatic adenocarcinoma by two pathologists. Ethical consent was approved by Sun Yat-sen University’s Committees for Ethical Review of Research involving Human Subjects. The characteristics and clinicopathological features of the patients are listed in Table 1. Tissue microarray containing 70 PCa specimens and 10 benign prostate hypertrophy (BPH) tissues, termed Cohort 2, were purchased from US Biomax (catalogue numbers PR808). Tissue microarray containing 18 PCa specimens, which was used to analyzed the expression of AR and LBCS, were purchased from US Biomax (catalogue numbers T195e).

**Table 1:** Association between LBCS expression and clinicopathological features of prostate cancer from Cohort 1

Characteristics	Cases (%)		*χ* ^*2*^	*P*-value
Total Cohort 1 Patients (N)	130			
Patients with complete clinical and follow up information (N)	130			
LBCS expression	Low	High		
Age (Year)				
≤70	30 (23)	39 (30)	2.502	0.114
>70	35 (27)	26 (20)		
Gleason Score				
6–7	27 (21)	50 (38)	16.851	0.000*
8–10	38 (29)	15 (12)		
Tumor stage				
T1–2	27 (21)	52 (40)	20.166	0.000*
T3–4	38 (29)	13 (10)		
Lymphnodes status N				
Negative	50 (38)	58 (45)	3.502	0.061
Positive	15 (12)	7 (5)		
Distant Metastasis M				
M0	55 (24)	59 (33)	1.140	0.286
M1	10 (25)	6 (18)		

*P<0.05 is considered significant

Median H-Score of LBCS was used as cut-off value for analysis

### Microarray analysis

The microarray screening for differently expressed lncRNAs in CRPC was reported previously [10]. All primary data in microarray analysis have been uploaded to the Gene Expression Omnibus and the accession numbers is GSE93929.

### The TCGA data mining

Patients’ clinical profiles in the TCGA prostate adenocarcinoma cohort [9] are available at https://cancergenome.nih.gov/. The expression of LBCS in PCa was obtained from TANRIC [12] (http://ibl.mdanderson.org/tanric/_design/basic/query.html). The TCGA prostate adenocarcinoma cohort comprising of 374 patients was used for the analysis. The survival of TCGA PCa patients were analyzed using GEPIA [13] (http://gepia.cancer-pku.cn/).

### RNA isolation and real time qPCR

Total RNA was extracted from cells using Trizol reagent (TaKaRa Biotechnology, Dalian, China) according to the manufacturer’s protocol. Total RNA was reverse transcribed with a PrimerScript RT-PCR kit (Takara Biotechnology, Dalian, China). Real time qPCR was conducted using a standard SYBR Green PCR kit (Roche, Upper Bavaria, Germany) protocol with a CFX real-time instrument (Bio-rad, Hercules, CA, USA). The relative expression was calculated using the 2^-∆∆Ct^ method. The transcription level of GAPDH was used as an internal control. All shRNA, siRNA and specific primers are listed in Additional file 1: Table S1 and Additional file 2: Table S2.

### In situ hybridization (ISH) and immunohistochemistry (IHC)

LBCS expression was also examined using ISH in formalin-fixed, paraffin-embedded (FFPE) samples, as previously described [14]. The IHC analyses and score calculation were conducted as described previously [14, 15]. Anti-AR antibodies (1:500, Santa Cruz, CA, USA) were used to detect the expression of AR in PCa tissues. The expression of LBCS and SOX2 in PCa specimens was quantified by using the histochemical score (H-score) as described previously [15]. The staining intensity was graded as follows: 0 (no staining), 1 (weak staining, light yellow for IHC, light blue for ISH), 2 (moderate staining, brown for IHC, moderate blue for ISH) and 3 (strong staining, brown red for IHC, strong blue for ISH). The intensity of staining was multiplied by the percentage of positive cells (0–100%), and the H-score (0–300) of each tissue was obtained for statistical analysis. The median H-score of all samples was used for cut-off values for high or low LBCS expression. The score of ISH and IHC in the FFPE samples was blindly quantified by two pathologists and the average H-score (0–300) of each tissue was obtained for statistical analysis. The probes were listed in Additional file 3: Table S3.

### Western blotting

Western blotting was performed as previously described [16, 17]. Primary antibodies specific to AR (1:200, Santa Cruz, CA, USA), PSA, OPRK1, TMPRSS2, GAPDH (1:1000, Cell Signaling Technology, MA, USA), hnRNPK (1:1000, Abcam, Massachusetts, USA) were used. The blots were then incubated with goat anti-rabbit or anti-mouse secondary antibody (Cell Signaling Technology, MA, USA) and visualized using enhanced chemiluminescence.

### Cell proliferation assay

The methyl thiazolyl tetrazolium (MTT; MTS, Promega, Madison, USA) colorimetric assay was used to detect cell viability. Cells were seeded in 96-well plates at a density of 2 × 10^3^ cells/well. Then, the absorbance was measured at a wavelength of 490 nm for 5 days using a SpectraMax M5 (Molecular Devices, CA, USA).

For the colony formation assay, the cells were seeded in a 96-well plate at a density of 500 cells per well. Seven days later, the clones were washed with 1× phosphate buffered saline (PBS) and stained with crystal violet for approximately 20 min. The clones were then imaged and quantified.

### Chemosensitivity assay

Cells were treated with different concentration of bicalutamide or R1881 (Sigma, St. Louis, MI, USA) for 120 h. The cell viability was measured using the same method as MTT assay. For calculation of half inhibition concentration (IC_50_), data were fitted in Graph Pad Prism 5 (Graph Pad Software Inc., San Diego, CA, USA) and dose-response curve was plotted using the equation log (inhibitor) vs. response- Variable slope. This is also called a four-parameter dose-response curve: Y=Bottom + (Top-Bottom)/ (1 + 10^ ((Log IC_50_-X) *HillSlope)) [14].

### RNA florescent in situ hybridization

The fluorescent in situ hybridization kit was purchased from Ribo Bio (Guangzhou, China) and the experiment is performed according to the manufacturer’s instruction and previously described [10], then visualized by a confocal microscope (Zeiss, Oberkochen, German). The CY3 labeled 18S probes were provided by the Ribo Bio (Guangzhou, China) and the LBCS probe was synthesized by Sangon (Shanghai, China). The sequences of LBCS and U6 probes were listed in Additional file 3: Table S3.

### Nuclear fraction

The cellular fraction was isolated as described previously [8]. Briefly, 10^7^ cells were harvested, resuspended in 1 mL of ice cold RNase-free PBS, 1 ml of buffer C1 (1.28 M Sucrose, 40 mM Tris-HCl, pH 7.5, 20 mM MgCl_2_, 4%Triton X-100) and 3 ml of RNase-free water, and incubated for 15 min on ice. Then cells were centrifuged for 15 min at 2500 rpm, the supernatant containing cytoplasmic constituent and the nuclear pellet were kept for RNA extraction.

### RNA pulldown and RIP assay

RNA pulldown was conducted as previously reported [14]. LBCS full-length sense and antisense sequences were prepared via in vitro transcription using a TranscriptAid T7 High Yield Transcription Kit (Thermo Scientific, USA). The RNA pulldown assay was performed using a Magnetic RNA-Protein Pull-down Kit (Thermo Scientific, USA) according to manufacturer’s instructions. The samples were separated using electrophoresis and LBCS-specific bands were identified using mass spectrometry and retrieved from a human proteome library. The RIP was performed as described previously [10] using the EZ-Magna RIPkit (Millipore, MA, USA). hnRNPK antibody (1:200, Abcam, Massachusetts, USA) were used. Normal rabbit IgG was used as a negative control.

### RNA isolation by RNA purification and chromatin immunoprecipitation (ChIP) assay

The RNA isolation by RNA purification was conducted using a Magna ChIRP RNA Interactome Kit (Millipore) according to the manufacturer’s instructions and as described previously [10]. ChIP was conducted using an EZ-Magna ChIP A/G kit (Millipore) according to manufacturer’s instructions and as previously reported [10] [14]. The method of RNA isolation by RNA purification and ChIP was detail in the Supplemental Materials and Methods section. The prodes used in RNA isolation by RNA purification are listed in Additional file 3: Table S3. The primers used in ChIP and RNA isolation by RNA purification real time qPCR are listed in Additional file 4: Table S4 and Additional file 5: Table S5.

### Statistical analyses

Quantitative data were presented as the means ± the standard deviation (SD) of three independent experiments. Differences between two groups were analyzed with the unpaired/paired Student’s t test (two-tailed tests), and one-way ANOVA followed by.

Dunnett’s multiple comparisons tests was performed when more than two groups were compared. Data of clinical analysis were shown as median with the interquartile range. The Mann-Whitney U test was used for independent samples when the population could not be assumed to be normally distributed. Pearson’s chi-square test was used to analyze the clinical variables. Spearman’s correlation analysis was performed to determine the correlation between two variables. Cumulative survival time was calculated using the Kaplan–Meier method and analyzed by the log-rank test. The best point cutoff value was used to define LBCS expression level (Low VS High) for analyzing TCGA cohort. The median H-score were used as cutoff value to define LBCS expression level (Low VS High) for analysis for cohort 2. A multivariate Cox proportional hazards model was used to estimate the adjusted hazard ratios and 95% confidence intervals, and to identify independent prognostic factors. All statistical analyses in this study were performed using SPSS 19.0 software. Actual *p*-values were provided in Additional file 6: Table S6. A *P* value < 0.05 was considered significant.

### Supplemental materials and methods

Supplemental Materials and Methods was provided as Additional file 7 and Additional file 8: Table S7.

## Results

### LBCS is markedly downregulated in CRPC cells

**Fig. 1:**
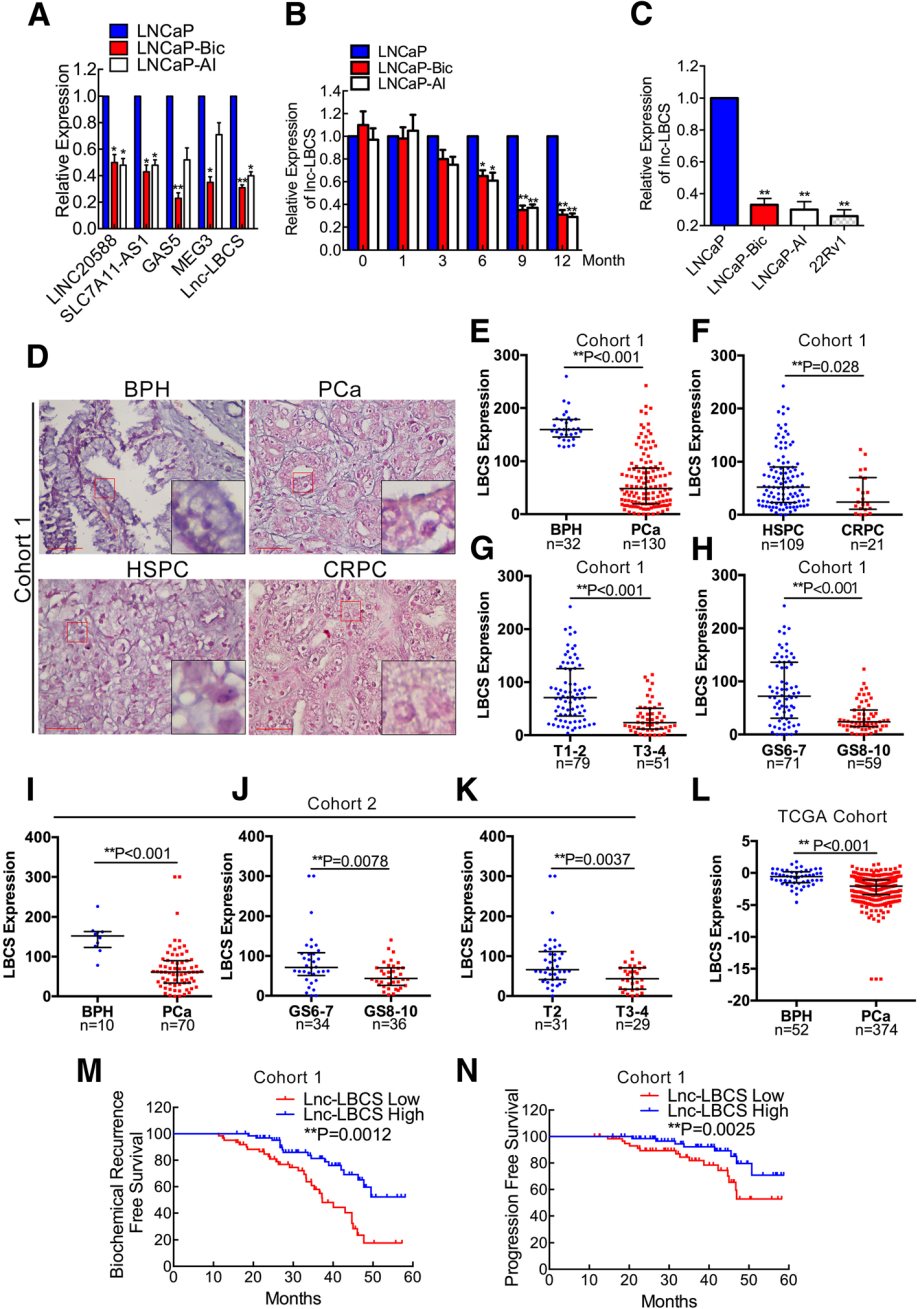
A CRPC related lncRNA LBCS associates with PCa clinical characteristics and good prognosis. **a** The differently expressed lncRNAs were validated by real time qPCR. **b** The expression of LBCS in LNCaP cells treated with either bicalutamide or androgen ablation at different point-in-time was detected by real time qPCR. **c** The expression of LBCS was detected in androgen dependent (LNCaP) and androgen independent (LNCaP-Bic, LNCaP-AI, 22Rv1) cell lines by real time qPCR. The results of real time qPCR were normalized to GAPDH and presented as the means ± SD of values obtained in three independent experiments. **d** Representative images of in situ hybridization (ISH) of LBCS expression (blue) in paraffin-embedded BPH (*n* = 32) and PCa (*n* = 130, 109 cases hormone sensitive and 21 cases of CRPC) prostate cancer tissue (Cohort 1). Red scale bars: 50 μm. **e-h** LBCS was detected between BPH and PCa, HSPC and CRPC, T1–2 and T3–4, Gleason Score (GS) 6–7 and 8–10 groups from cohort 1 by ISH. ISH of LBCS expression was quantified by the expression score (0–300). Patients with unavailable information was excluded for analysis. **i-k** LBCS was detected between BPH (*n* = 10) and PCa (*n* = 70), Gleason Score (GS) 6–7 and 8–10, T2 and T3–4 groups from cohort 2 by ISH. ISH of LBCS expression was quantified by the expression score (0–300). The whiskers indicate median ± interquartile in the plots. **l** The expression was LBCS was analyzed between 52 cases of BPH and 374 cases of PCa from TCGA. The whiskers indicate median ± interquartile in the plots. **m-n** The biochemical recurrence-free survival and progression-free survival rates of the 130 PCa patients from cohort 1 were compared in the LBCS-low and LBCS-high groups. Median H-Score was used as cut off value in the survival analysis. The patients with complete clinical and follow up information were adopted for survival, univariate and multivariate analysis. (See also Additional file 9: Figure S1) *p < 0.05, **p < 0.01

In our previous study, we had reported the differently expressed lncRNAs in LNCaP cells and its two castration resistant sublines, which we name as LNCaP-AI and LNCaP-Bic [10]. We identified that HOXD-AS1 was overexpressed in CRPC cells previously, and we focus on the downregualted lncRNAs in this study. We found a novel lncRNA termed LBCS that was among the most downregulated lncRNAs in CRPC cells from the previously reported microarray results [10]. Recent study found that LBCS inhibits self-renewal, chemoresistance and tumor initiation of bladder cancer stem cells by guiding the hnRNPK-EZH2 complex to repress the expression of SOX2 [14]. However, the biological function and mechanism of LBCS in PCa progression remain unknown. Then we confirmed that LBCS was among the most downregulated lncRNAs in CRPC cell lines by real time qPCR (Fig. 1a). Additionally, we observed that the expression of LBCS decreased gradually with prolonged androgen ablation (Fig. 1b), and LBCS was also lowly expressed in androgen independent 22Rv1 cells compared with LNCaP cells (Fig. 1c).

### LBCS associates with PCa clinical characteristics and good prognosis

To investigate whether LBCS was involved in clinical PCa progression, we detected and analyzed LBCS expression in three independent cohorts of PCa specimens. Firstly, we analyzed its expression in Cohort 1 containing 130 PCa and 32 benign prostate hypertrophy (BPH) tissues from our hospital by in situ hybridization (ISH). We found that the LBCS expression was significantly lower in PCa compared with BPH tissues (Fig. 1d-e). Interestingly, the expression of LBCS was also markedly down-regualted in CRPC patients compared with hormone sensitive prostate cancer (HSPC) patients (Fig. 1d, f), in T3–4 tumors compared with T1–2 (Fig. 1g), in tissues with Gleason Score of 8–10 compared with 6–7 (Fig. 1h, Additional file 9: Figure S1A-B). Further analysis revealed that LBCS expression was correlated closely with T stage and Gleason Score in Cohort 1 (Table 1). To confirm the results, we evaluated the expression of LBCS by ISH from a Cohort 2, which including 70 cases of PCa and 10 cases of BPH tissues. Consistent with Cohort 1, we observed that the LBCS expression was significantly downregulated in PCa compared with BPH tissues (Fig. 1i), in tissues with Gleason Score of 8–10 compared with 6–7 (Fig. 1j), in T3–4 tumors compared with T2 (Fig. 1k, Additional file 9: Figure S1C-D). Meanwhile, LBCS expression was also associated with T stage and Gleason Score from Cohort 2 (Additional file 10: Table S8). In order to further confirm the clinical significance of LBCS in PCa patients, we analyzed a large-scale RNA-seq dataset and the corresponding clinical information from The Cancer Genome Atlas (TCGA) [9]. A total of 374 cases of PCa and 52 cases of BPH tissues were included. We found that the expression of LBCS was significantly downregulated in PCa compared with BPH tissues (Fig. 1l). The data suggest that LBCS may play a key role in PCa initiation and progression.

We then explore whether LBCS expression is associated with prognosis of PCa patients. Kaplan-Meier survival analysis of Cohort 1 showed that low LBCS-expressing PCa patients had significantly shorter biochemical recurrence-free survival (BRFS) and progression-free survival (PFS) (*P* = 0.0012, 0.0025, respectively. Figure 1m-n). Multivariate analyses revealed that LBCS expression was independent prognostic factor for BRFS in PCa patients (*P* = 0.04, Table 2), but not PFS (Additional file 11: Table S9). Additionally, analyzing the survival of TCGA profiles by GEPIA [13], we observed that high LBCS expression was associated with longer overall survival and disease-free survival, though it was not statistically significant (Additional file 9: Figure S1E-F). These findings clearly demonstrate the potential of LBCS as a marker of good prognosis in PCa.

**Table 2:** Univariate and multivariate analysis of factors associated with biochemical recurrence-free survival in prostate cancer cohort 1

Variable	Univariate			Multivariate		
	HR	95% CI	*p*	HR	95% CI	*p*
Age, years (> 70/≤70)	0.958	0.539–1.704	0.884			NA
Gleason Score (8–10/6–7)	2.171	1.221–3.859	**0.008**	1.651	0.866–3.146	0.128
Tumor stage (T3–4/T1–2)	2.148	1.203–3.837	**0.014**	1.397	0.739–2.641	0.304
Nodal metastasis (N1/N0)	2.169	1.072–4.386	**0.031**	2.105	1.004–4.415	0.05
Distant metastasis (M1/M0)	2.104	0.878–5.041	0.095			NA
LBCS (high/low)	0.348	0.191–0.635	**0.001**	0.477	0.235–0.967	**0.040**

Univariate and multivariate analysis. Cox proportional hazards regression model. Variables associated with survival by univariate analyses were adopted as covariates in multivariate analyses. Significant *P*-values are shown in bold font. HR > 1, risk for death increased; HR < 1, risk for death reduced. Median H-Score of LBCS was used as cut-off value for analysis

### LBCS restores the castration sensitivity of CRPC cells

**Fig. 2:**
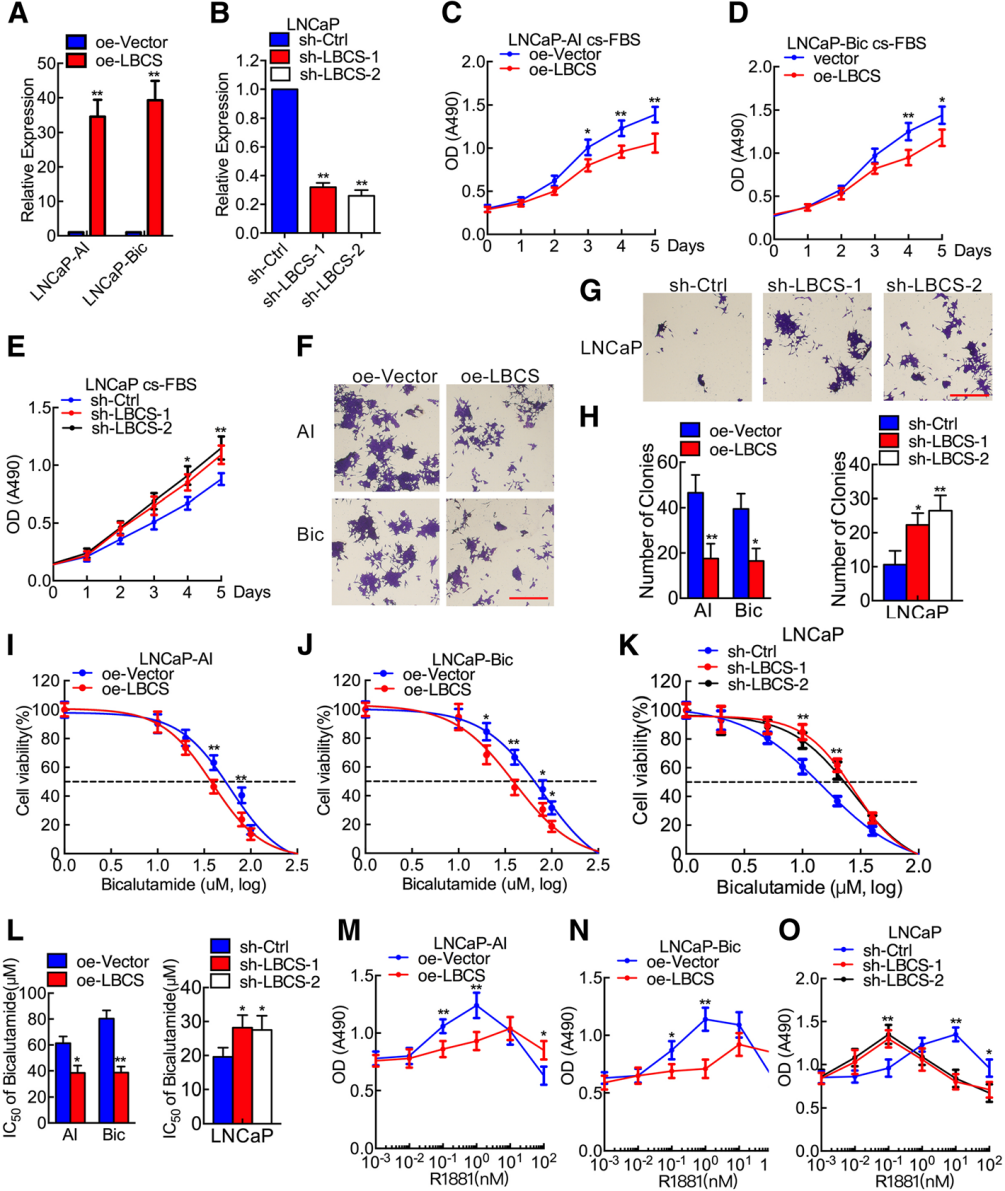
LBCS restores the castration sensitivity of CRPC cells. **a**-**b** Efficiency of stable overexpression of LBCS in LNCaP-AI and LNCaP-Bic, or knockdown of LBCS by two shRNAs in LNCaP cells was verified by real time qPCR. **c**-**e** The effect of LBCS overexpression on viability in LNCaP-AI and LNCaP-Bic cells, or knockdown on LNCaP cells in androgen deprived cultural medium. Cs-FBS indicates charcoal stripped fetal bovine serum. **f**-**h** The effect of LBCS overexpression on colony formation in LNCaP-AI and LNCaP-Bic cells, or knockdown on LNCaP cells in androgen deprived cultural medium. Scale bar: 200 μm. **i**-**k** LBCS overexpressed LNCaP-AI and LNCaP-Bic cells, LBCS depleted LNCaP cells were treated with different concentration of bicalutamide for 120 h and cell viability was analyzed by MTT assay. **l** Calculation of IC_50_ of bicalutamide in LNCaP-AI, LNCaP-Bic and LNCaP cells, four parameter logistic curve (best-fit solution, nonlinear regression dynamic fitting) and normality tests are used (Graph Pad Prism 6). **m**-**o** LBCS overexpressed LNCaP-AI and LNCaP-Bic cells, LBCS depleted LNCaP cells were treated with different concentration of R1881 for 120 h and cell viability was analyzed by MTT assay. The results are presented as the means ± SD of values obtained in three independent experiments. (See also Additional file 12: Figure S2) *p < 0.05, **p < 0.01

To explore the biological function of LBCS in PCa progression, we first stable overexpressed or knocked down LBCS expression in PCa cells by lentivirus. Real time qPCR showed that LBCS was remarkably upregulated in LNCaP-Bic, LNCaP-AI cells and downregulated in LNCaP cells, as compared with respective control (Fig. 2a-b). LBCS overexpression decreased proliferation of castration resistant LNCaP-Bic and LNCaP-AI in androgen ablated medium (Fig. 2c-d), and LBCS depletion promoted the proliferation of androgen sensitive LNCaP cells under castration condition (Fig. 2e). Consistent with cell growth results, LBCS overexpressed CRPC cells formed significantly fewer and smaller colonies whereas LBCS knocked down LNCaP cells formed more and bigger colonies, as compared with control cells (Fig. 2f-h). Additionally, we found that LBCS overexpression dramatically increased cell population at G0/G1 phase, while reduced cell population at S phase (Additional file 12: Figure S2A-B) in LNCaP-AI and LNCaP-Bic cells. Conversely, LBCS silencing significantly increased cell population at S phase in LNCaP cells, as detected by flow cytometry. These data indicate that LBCS inhibits cell viability of PCa cells under androgen deprived condition.

Bicalutamide is the first-line drug of ADT in PCa. However, PCa cells no longer responds to bicalutamide after its progression to CRPC cells. According to our previous study, LNCaP-AI and LNCaP-Bic cell lines are resistant to bicalutamide treatment [10]. As a result, we investigated whether LBCS regulated the resistance to bicalutamide in PCa cells. Interestingly, overexpression of LBCS restored the sensitivity of LNCaP-Bic and LNCaP-AI to bicalutamide and produced a lower bicalutamide IC_50_ compared with that of the control cells (Fig. 2i-j, l). In contrast, LBCS downregulation promoted the resistance of bicalutamide therefore increased the bicalutamide IC_50_ in LNCaP cells (Fig. 2k-l). Additionally, compared with control cells, the caspase 3/7 activity was upregulated in LBCS overexpressed whereas downregulated in LBCS depletion cells upon treating with bicalutamide (Additional file 12: Figure S2C-D). Then we explore the effect of LBCS on the sensitivity of androgen stimulation. We detected cell viability under different R1881 stimulation as high concentration of androgen inhibits prostate cancer growth. Interestingly, we observed that LBCS overexpression significantly decreased the viability of CRPC cells under 10^− 1^ ~ 10 nmol R1881 treatment, in contrast, the viability of CRPC cells was not inhibited by higher concentration of R1881 of 10^2^ nmol, as compared with the control group (Fig. 2m-n). On the other hand, we also found that LBCS depletion sensitized LNCaP cells to a lower R1881 concentration but inhibited its viability drastically under 10~10^2^ nmol of R1881 (Fig. 2o). As a result, our data showed that LBCS overexpression inhibited, whereas LBCS depletion promoted, the sensitivity of androgen treatment in PCa cells. Taken together, we demonstrated that LBCS sensitized CRPC cells to castration condition, and inhibited androgen stimulation response in PCa cells.

### LBCS inhibits AR protein translation and restrains AR signaling activation

**Fig. 3:**
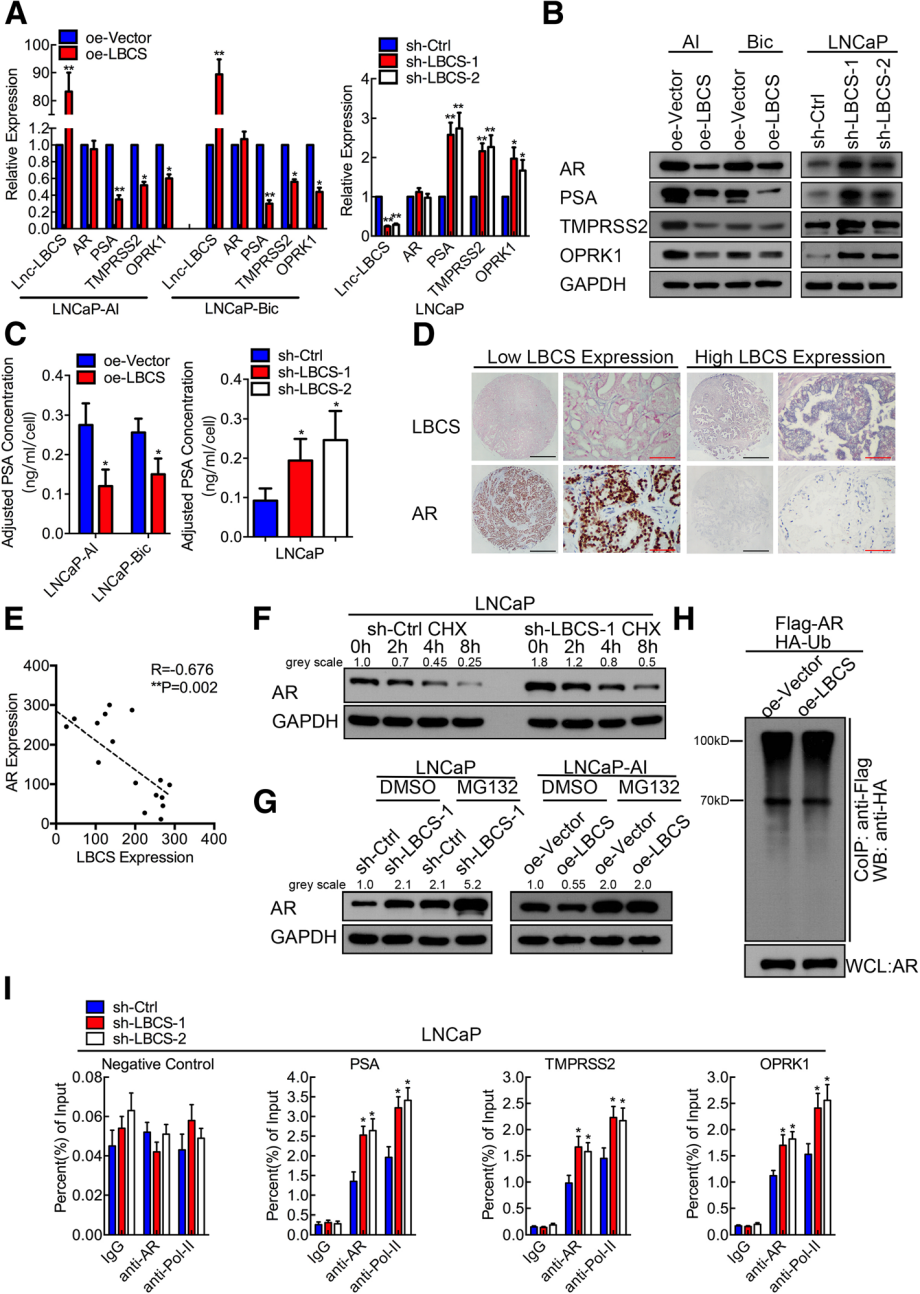
LBCS inhibits AR protein translation and restrains AR signaling activation. **a** The AR and its target genes were detected in LBCS overexpressed LNCaP-AI and LNCaP-Bic cells, LBCS knockdown LNCaP cells by real time qPCR. The results were normalized to GAPDH and presented as the means ± SD of values obtained in three independent experiments. **b** The protein level of AR and its target genes were detected in LBCS overexpressed LNCaP-AI and LNCaP-Bic cells, LBCS knockdown LNCaP cells by western blotting. **c** The concentration of PSA was validated in cultural supernant of LBCS overexpressed LNCaP-AI and LNCaP-Bic cells, LBCS knockdown LNCaP cells. The PSA concentration was adjusted by cell count of each group. The results are presented as the means ± SD of values obtained in three independent experiments. **d**-**e** LBCS was detected by ISH and AR was detected by ICH in 18 cases of PCa tissue. H-Score was calculated then analyzed by Spearman’s correlation analysis. Representative images of ISH and image were displayed. Black scale bar: 500 μm, red scale bar: 50 μm. **f** LBCS depletion LNCaP and control cells were treated with cycloheximide for 2, 4 and 8 h. The AR protein expression was detected by western blotting. The grey scale of the blot was analyzed by Image J. **g** LBCS depletion LNCaP and overexpressed LNCaP-AI cells with respective control cells were treated with either DMSO or MG132 for 8 h. The AR protein expression was detected by western blotting. The grey scale of the blot was analyzed by Image J. **h** Flag-AR and HA-ubiquitin vectors were co-transfected to either LBCS overexpressed or control 293 T cells. CoIP was conducted by anti-flag (AR) antibody then the product was detected using anti-HA (ubiquitin) antibody. AR expression of whole cell lysates (WCL) was detected as loading control of each group. **i** ChIP analysis of IgG, AR, and RNA polymerase-II status of AR target genes in LNCaP cells after LBCS knockdown. The values are normalized to input and presented as the means ± SD. (See also Additional file 13: Figure S3) *p < 0.05, **p < 0.01

Considering that LBCS functionally affects castration resistance of PCa cells, we then explored whether LBCS regulates AR signaling pathway. We found that the expression of AR downstream genes including PSA, TMPRSS2 and OPRK1 were significantly downregulated upon LBCS overexpression in CRPC cells, but the mRNA expression of AR showed no significant change (Fig. 3a). Conversely, these AR targets were upregulated in LBCS knockdown LNCaP cells (Fig. 3a). Interestingly, we found that AR protein and its target genes was downregulated in CRPC cells with overexpressed LBCS, whereas upregulated in LBCS depleted LNCaP cells by western blotting (Fig. 3b). Additionally, we discovered that the PSA level of cultural medium was significantly decreased in LBCS overexpression CRPC cells, while increased significantly in LBCS knockdown LNCaP cells, as detected by chemiluminescence (Fig. 3c). To further validate the relationship between LBCS and AR, we evaluated the expression of LBCS and AR in 18 cases of prostate cancer tissue by ISH and immunohistochemistry (IHC) (Fig. 3d). Concordantly, the expression of LBCS and AR protein was negatively correlated in prostate cancer specimen (*P* = 0.002, *R* = − 0.676, Fig. 3e). Next, we investigated how LBCS regulate AR expression at protein level. Firstly, to determine whether LBCS stabilizes AR protein, we treated LNCaP with cycloheximide (CHX), an inhibitor of protein biosynthesis. Western blot analysis showed that the half-life of AR protein was about 3.5 h in control cells, which did not change a lot in LBCS knockdown LNCaP cells (Fig. 3f), indicating that AR degraded at equal proportion between groups (Additional file 13: Figure S3). Moreover, we treated cells with proteasome inhibitor MG132. We found that MG132 dramatically increased AR protein level, confirming successful blockade of proteasome-mediated protein degradation (Fig. 3g). Furthermore, LBCS downregulation increased AR protein level in the control-treated cells, while more significant difference was shown in the cells pretreated with MG132. On the other hand, MG132 treatment depleted the inhibition of AR protein by LBCS overexpression, as compared with control-treated cells (Fig. 3g). These data indicate LBCS inhibited AR translation instead of proteasome-mediated AR degradation. Finally, to examine whether LBCS affects AR ubiquitination, we performed co-transfection of FLAG-AR, HA-ubiquitin, LBCS or control vectors into 293 T cells. Western blot analysis of whole-cell lysate (WCL) confirmed AR protein expression in all experimental conditions. Cell lysates were then subjected to immunoprecipitation (IP) by an CoIP using an anti-FLAG (AR) antibody followed by western blotting using an anti-HA (ubiquitin) antibody confirmed that LBCS did not affect the ubiquitination of AR protein (Fig. 3h). Our results showed that LBCS knockdown promoted AR protein translation, instead of ubiquitination nor proteasome-mediated degradation. Additionally, we further validated whether knockdown LBCS activates AR signaling directly. We performed ChIP-qPCR by anti-AR antibody on several known AR target genes using site-specific primers. Our data confirmed that LBCS downregulation indeed increased AR and Pol-II recruitment to promotors of PSA, TMPRSS2 and OPRK1, leading to AR signaling activation (Fig. 3i). Collectively, our data demonstrated that LBCS is a novel suppressor of AR protein translation and AR signaling activation.

### LBCS interacts with AR mRNA directly

**Fig. 4:**
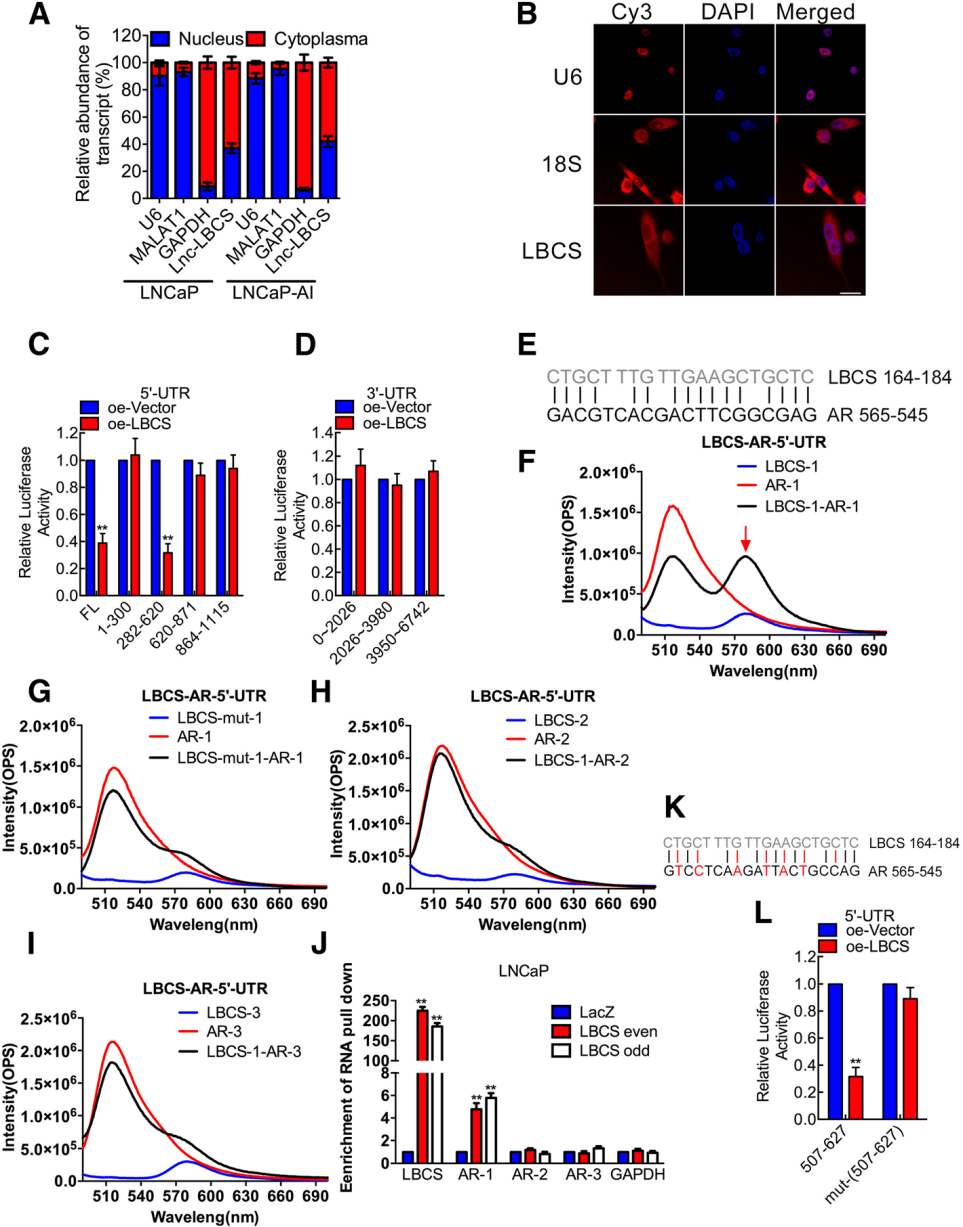
LBCS interacts with AR mRNA directly. **a** Nuclear fraction experiment and real time qPCR detected the abundance of LBCS in the nucleus and cytoplasm. GAPDH is the positive control for cytoplasm, and MALAT1 and U6 is the positive control for nucleus. The results are presented as the means ± SD of values obtained in three independent experiments. **b** The subcellular distribution of LBCS was visualized by RNA Fluorescent in situ hybridization (FISH) in LNCaP cells. 18S was the positive control for cytoplasm, and U6 was the positive control for the nucleus. Scale bar: 100 μm. **c**-**d** Luciferase vectors were constructed containing full length 5′-UTR, different segments of 5′-UTR and 3′-UTR of AR mRNA. The luciferase activity was detected by either co-transfecting control vector or LBCS overexpression vector. The results are presented as the means ± SD of values obtained in three independent experiments. **e** Potential AR mRNA-LBCS interacting sites were predicted and illustrated. **f**-**i**) FRET was performed using a 1:1 mixture of in vitro synthesized LBCS and different AR 5′-UTR RNA regions. **j** RNA isolation by RNA purification experiment was conducted using LNCaP cells, the different segments of AR 5′-UTR was detected by real time qPCR. GAPDH was detected as a non-specific control. The values are normalized to the negative control LacZ probe and presented as the means ± SD. **k** Site-directed mutagenesis was conducted on the LBCS interacting site of AR 5′-UTR, the letters in red indicates the mutant base pairs. **l** The effect of site-directed mutagenesis on the interaction between LBCS and AR mRNA was detected by luciferase assay. The results are presented as the means ± SD of values obtained in three independent experiments. *p < 0.05, **p < 0.01

The subcellular localization of lncRNA is associated closely with its biological function [7]. The cellular fractionation assays and RNA fluorescence in situ hybridization (RNA FISH) showed that LBCS was distributed in both nuclear and plasma of PCa cells, but mainly in plasma (Fig. 4a-b), suggesting that LBCS might exert a post-transcriptional regulation function. Previous studies reveal that the element of translational regulation mainly locates in 5’UTR region [18]. To address this, we performed luciferase assay by cloning the full length (FL) 5′-UTR and different segments of 5′-UTR and 3′-UTR as control to the luciferase vector. Interestingly, we found that the luciferase activity was decreased significantly when co-transfecting LBCS with psiCHECK2–5′-UTR, but not the vector containing 3′-UTR (Fig. 4c-d). Furthermore, we identified that LBCS markedly suppressed the luciferase activity of 5′-UTR 282–620 region, but not 1–300, 620–871 and 864–1115 regions (Fig. 4c). By sequence prediction, we found three potential LBCS-AR binding regions (termed AR1–3, Fig. 4e, Additional file 14: Table S10). To confirm the precise binding sites, fluorescence resonance energy transfer (FRET) was performed using in vitro synthesized LBCS and AR 5′-UTR RNA regions. Upon excitation at 460 nm, the emission at 580 nm increased, while the signal at 520 nm decreased in the LBCS (164-184 nt)/AR group, but not other groups, compared with that of the control RNA/AR (Fig. 4f-i). These data indicated that LBCS 164–184 nt region directly interacts with the 5′-UTR 545–565 nt region of AR (AR1), but not other regions (AR2, AR3). Moreover, we conducted an RNA Isolation by RNA Purification experiment then detected the enrichment of specific AR mRNA regions by real time qPCR in LNCaP cells. Interestingly, we found that 5′-UTR containing AR1 regions, but not other regions, was enriched by LBCS probes, as compared with negative control LacZ probes (Fig. 4j). Meanwhile, we generated luciferase vector containing mutant AR1 region by site directed mutagenesis (Fig. 4k). We found that LBCS inhibited luciferase activity of wild type AR1 region significantly but not the mutant region (Fig. 4l). Taken together, our results supported that LBCS interacted directly with AR mRNA to inhibit its translation.

### LBCS binds and recruits hnRNPK to AR mRNA to inhibit AR translation in PCa

**Fig. 5:**
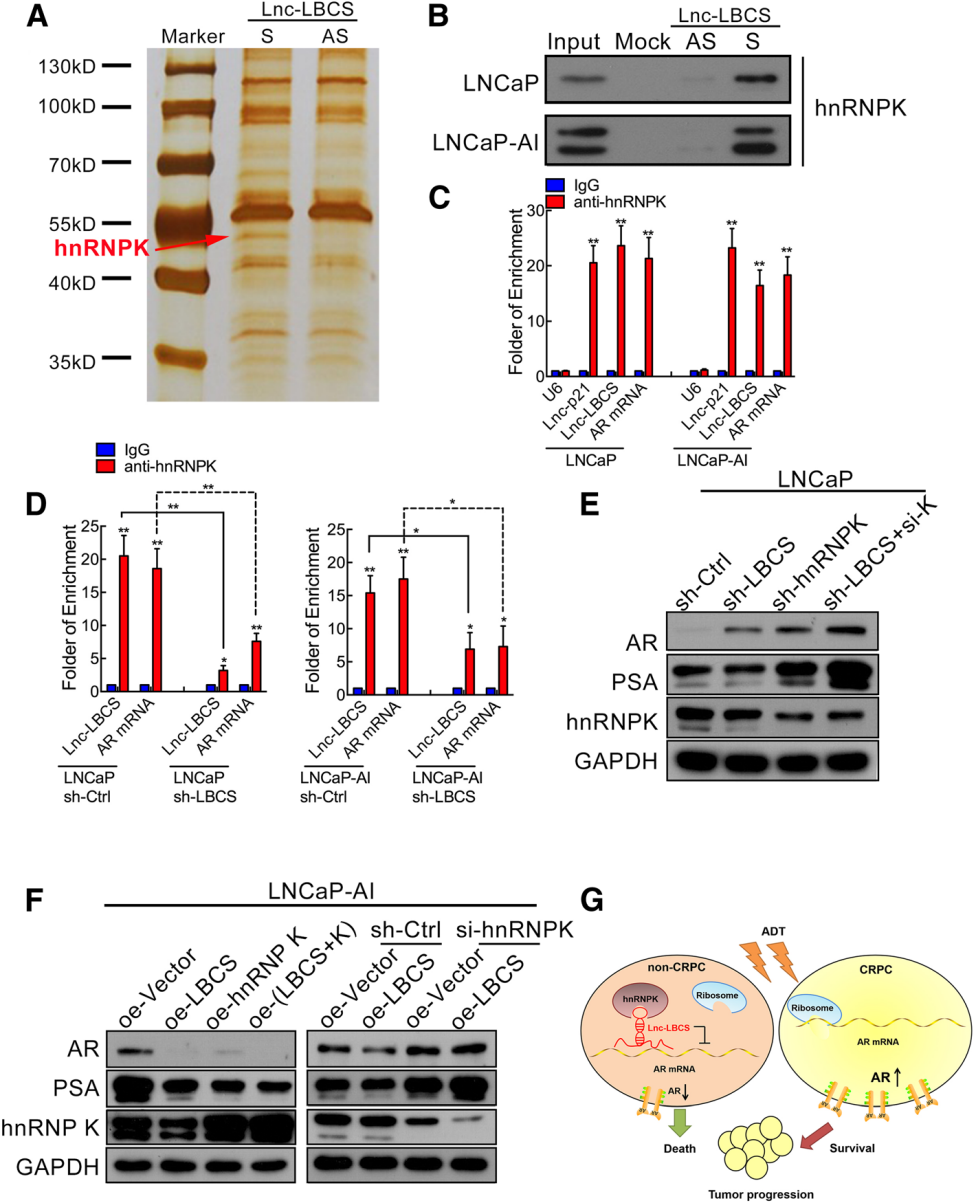
LBCS binds and recruits hnRNPK to AR mRNA to inhibit AR translation in PCa. **a** RNA pulldown assay was performed using LBCS sense and antisense RNAs incubated with cell lysates of LNCaP cells, followed by silver staining. The red arrow indicates hnRNPK. **b** The interaction between LBCS and hnRNPK was confirmed by RNA pulldown followed by western blotting in LNCaP and LNCaP-AI cells. **c** Real time qPCR analysis of LBCS and AR mRNA in RNA immunoprecipitation (RIP) assay of LNCaP and LNCaP-AI cells using anti-hnRNPK, RNA enrichment was determined relative to the non-immuned IgG control. U6 was used as a non-specific control. Lnc-p21 was used as a positive control. The results are presented as the means ± SD of values obtained in three independent experiments. **d** RIP assay using anti-hnRNPK was performed in either LBCS knockdown or control LNCaP and LNCaP-AI cells, the enrichment of LBCS and AR mRNA was detected by real time qPCR. The results are presented as the means ± SD of values obtained in three independent experiments. **e** The effect of combined knockdown of LBCS and hnRNPK on the expression of AR in LNCaP cells, as compared with silencing each of LBCS or hnRNPK, or control shRNA, as assessed by western blotting. GAPDH were used as internal control. **f** The effect of combined overexpression of LBCS and hnRNPK on the expression of AR in LNCaP-AI cells, and the effect of knockdown hnRNPK on AR expression in LBCS overexpressed or control cells. GAPDH were used as internal control. **g** A schematic model of the mechanism underlying the role of LBCS in castration resistance of prostate cancer. *p < 0.05, **p < 0.01

LncRNA usually exerts its regulatory function by binding to proteins [19]. So, we applied an RNA pull-down assay to further elucidate the mechanism of LBCS in PCa cells. One overtly differential band around 55kD appeared by silver staining and was identified as heterogeneous nuclear ribonucleoprotein K (hnRNPK) by mass spectrometry (Fig. 5a). Moreover, we confirmed the interaction of LBCS with hnRNPK using western blotting (Fig. 5b). The result is consistent with our findings in the recent study of bladder cancer [14]. Interestingly, a previous report found that hnRNPK is critical suppressor of AR translation, and the expression of hnRNPK and AR is negatively correlated in prostate cancer [20]. However, the detail mechanism of how hnRNPK binds to AR is unclear. To investigate whether LBCS act as a scaffold for hnRNPK-AR interaction, we performed a RNA immunoprecipitation (RIP) and found a significant enrichment of both LBCS and AR mRNA by hnRNPK antibody compared with IgG (Fig. 5c). Furthermore, the enrichment of AR mRNA by hnRNPK antibody was significantly decreased in LBCS downregulated LNCaP and LNCaP-AI cells, as compared with negative control cells (Fig. 5d), suggesting that the interaction between AR mRNA and hnRNPK is dependent on LBCS. Additionally, we further confirm that the suppression of AR by LBCS is in hnRNPK dependent manner by western blotting (Fig. 5e-f). We observed that combined knockdown of LBCS and hnRNPK increased the level of AR more drastically than knockdown either LBCS or hnRNPK (Fig. 5e). Conversely, combined overexpression of LBCS and hnRNPK showed stronger inhibition of AR than overexpression LBCS or hnRNPK alone (Fig. 5f). Interestingly, knockdown of hnRNPK completely abolished the inhibition of AR protein mediated by LBCS (Fig. 5f). PSA was detected representing the change of AR signaling in each experiment, indicating that the AR pathway activation was mediated by LBCS and hnRNPK. In summary, these results suggested that LBCS directly interacted with hnRNPK and recruited it to inhibit AR translation in PCa.

## Discussion

Emerging evidence show that lncRNAs play important regulatory roles in tumorigenesis and cancer drug resistance [21–23]. In this study, we first demonstrated that lncRNA LBCS is significantly downregulated in PCa and CRPC cells and cancer tissue, and correlated with tumor stage, Gleason Score and prognosis. Moreover, we propose a novel working model wherein LBCS suppressed the castration resistance of PCa by guiding hnRNPK to inhibit AR translation, which consequently attenuated PCa progression and castration resistance. These findings indicate that LBCS acts as a tumor suppressor in PCa progression and castration resistance and could be considered as a potential prognostic bio-marker and therapeutic target for PCa.

Recent studies reveal that lncRNAs, for instance, HOXD-AS1 [10], HOTAIR [24], PCGEM1 [11, 25], and ARLNC1 [26], regulate PCa progression through various mechanisms. However, the mechanism underlying how lncRNAs regulate AR signaling remains elusive. In this study, we identified lncRNA LBCS as a novel AR translational suppressor that inhibits progression of CRPC. Firstly, we identified lncRNA LBCS was downregulated significantly in CRPC cell models and tumor specimens by transcriptome microarray. Moreover, LBCS expression was negatively correlated with T stage and Gleason score in two different cohorts, while it was positively associated with better biochemical recurrence-free survival and progression free survival. Functionally, LBCS inhibited the viability of CRPC cells in different castration condition, the sensitivity of prostate cancer cells to androgen stimulation even under low concentration. These results indicated the tumor suppressor role of LBCS in progression and castration resistance of PCa, and LBCS might server as a marker of PCa progression and prognosis.

AR is reported to play the most important role and server as first-line therapy target in the PCa [27, 28]. Accumulating evidence finds that AR protein is elevated in approximately 80~90% CRPC patients, leading to AR signaling activation in the milieu of low androgen therefore provides sufficient growth signaling for PCa cells [3, 4]. In this paper, we show that LBCS suppressed the AR-activated gene expression by directly inhibiting the protein translation of AR. To our knowledge, lncRNAs regulate AR signaling through different mechanisms. For instance, HOTAIR inhibits E3-ubiquintin mediated AR degradation by binding with AR [24], while ARLNC1 stabilizes AR mRNA through specific RNA-RNA interaction [26]. Although recent studies describe that lncRNAs participated regulating protein translation [29], to date, whether lncRNA participates in the regulation of AR translation is still unknown. In the present study, we reported a novel lncRNA LBCS which inhibited the protein expression of AR and subsequent pathway activation. Further investigation confirmed that LBCS regulated AR through suppressing its translation directly. Thus, our findings revealed a mechanism that the AR translational was increased directly while the downregulating of LBCS during hormone sensitive prostate cancer (HSPC) progression to CRPC.

LncRNAs usually interacts with protein to serves as molecular scaffold or decoy, which guide protein to specific genetic loci by mediating RNA-RNA interaction [7]. HnRNPK is an essential RNA- and DNA-binding protein that plays a critical role in several cancers [30]. It has been proven a critical inhibitor of AR translation by interacting with AR mRNA [20], however, a detailed mechanism remains unknown. Here, we described that LBCS interacted directly with hnRNPK and then inhibited AR expression by forming a LBCS-hnRNPK-AR mRNA complex. We also elucidated that LBCS guided hnRNPK to exert its function by directly interacting with the 5′-UTR region of AR mRNA. Moreover, the inhibitive effect of LBCS on AR translation was in hnRNPK dependent manner. Our previous study reportes that LBCS binds and recruits hnRNPK-EZH2 complex to inhibit the expression of SOX2 in the nuclei of bladder cancer stem cells. However, in this present study, EZH2 was not detected to bind with LBCS in PCa. We found that LBCS and hnRNPK located in both nuclear and plasm of PCa cells, but EZH2 only located in the nuclear. So we think this may resulted from the characteristics of lncRNA itself, the diverse mechanisms a lncRNA could present in different circumstances and diseases [31, 32]. LncRNAs participate in translational regulation through different mechanisms. A recent study showed that lncRNA-TRMP inhibits the translation of p27 by binding competitively with PTBP1 thus promoting the proliferation of tumor cells [29]. Interestingly, a similar translational suppression mechanism is also observed. The non-coding tre-RNA binding with the hnRNPK to inhibit the translation of E-Cadherin, promoting the epithelial to mesenchymal transition of breast cancer [33]. Collectively, our finding reveals a novel epigenetic regulation mechanism of AR by LBCS in PCa. During the progression of androgen-dependent PCa to CRPC, the LBCS-hnRNPK-AR mRNA complex was weakened by LBCS downregulation, therefore increasing the protein translation of AR, which subsequently enhancing AR signaling and sustaining the proliferation of PCa cells under androgen ablation. Thus, LBCS might be a promising target for improving the treatment of CRPC (Fig. 5g).

In summary, it is our novel discovery that LBCS inhibits the castration resistance of PCa by decreasing the translation of AR through guiding hnRNPK to interacting directly with the 5′-UTR of AR mRNA. Therefore, our findings provide insight into LBCS might be a prognostic marker for PCa, as well as in the development of novel treatment against CRPC.

## Additional files

Additional file 1: **Table S1.** The sequences of siRNAs and shRNAs. (DOCX 13 kb)

Additional file 2: **Table S2.** The primers used in real time qPCR. (DOCX 13 kb)

Additional file 3: **Table S3.** The probes used in this article. (DOCX 14 kb)

Additional file 4: **Table S4.** The primers used in ChIP-real time qPCR. (DOCX 13 kb)

Additional file 5: **Table S5.** The primers used for RNA isolation by RNA purification-real time qPCR. (DOCX 13 kb)

Additional file 6: **Table S6.** Actual *p*-values of all figures. (DOCX 16 kb)

Additional file 7: Supplemental Material and Method. (DOCX 17 kb)

Additional file 8: **Table S7.** The primers used in gene clone (DOCX 14 kb)

Additional file 9: **Figure S1.** The clinical significance of lnc-LBCS in prostate cancer. (A-B) Lnc-LBCS was detected between N0 and N1, M0 and M1 groups from cohort 1 by ISH. ISH of lnc-LBCS expression was quantified by the expression score (0–300). Patients with unavailable information was excluded for analysis. The whiskers indicate median ± interquartile in the plots. (C-D) Lnc-LBCS was detected between N0 and N1, M0 and M1 groups from cohort 2 by ISH. ISH of lnc-LBCS expression was quantified by the expression score (0–300). Patients with unavailable information was excluded for analysis. The whiskers indicate median ± interquartile in the plots. (E-F) The overall survival and disease-free survival rates of the 492 PCa patients from TCGA were analyzed by GEPIA. *p < 0.05, **p < 0.01. (JPG 1018 kb)

Additional file 10: **Table S8.** Association between lnc-LBCS expression and clinicopathological features of prostate cancer from Cohort 2. (DOCX 15 kb)

Additional file 11: **Table S9.** Univariate and multivariate analysis of factors associated with progression-free survival in prostate cancer Cohort 1. (DOCX 15 kb)

Additional file 12: **Figure S2.** LBCS restores the castration sensitivity of CRPC cells. (A-B) LBCS was overexpressed in LNCaP-AI and LNCaP-Bic cells and knocked down in LNCaP cells, then cell cycles were analyzed by flow cytometry. (C-D) The caspase 3/7 activity was measured in lnc-LBCS overexpressed LNCaP-AI and LNCaP-Bic cells, and lnc-LBCS knockdown LNCaP cells treated with bicalutamide. *p < 0.05, **p < 0.01. (JPG 922 kb)

Additional file 13: **Figure S3.** The illustration of the change of AR grey scale after treated with cycloheximide for different hours in either LBCS knockdown or control group. (TIFF 93 kb)

Additional file 14: **Table S10.** The predicted LBCS-AR mRNA interacting sequences and oligos used for FRET. (DOCX 14 kb)

## Data Availability

The primary data in microarray analysis have been uploaded to the Gene Expression Omnibus and the accession numbers is GSE93929. The rest of datasets used and analysed during the current study are available from the corresponding author on reasonable request.

## Abbreviations

3’UTR: 3′ untranslated region
5’UTR: 5′ untranslated region
ADT: Androgen deprivation therapy
AR: Androgen receptor
BPH: Benign prostate hypertrophy
BRFS: Biochemical recurrence-free survival
ChIP: Chromatin immunoprecipitation
CHX: Cycloheximide
CRPC: Castration resistant prostate cancer
FRET: Fluorescence resonance energy transfer
hnRNPK: heterogeneous nuclear ribonucleoprotein K
HSPC: Hormone sensitive prostate cancer
IC_50_: Half maximal inhibitory concentration
IHC: Immunohistochemistry
IP: Immunoprecipitation,
ISH: In situ hybridization
LBCS: Low expressed in bladder cancer stem cells
LncRNA: Long noncoding RNA
PCa: Prostate cancer,
PFS: Progression-free survival
PSA: Prostate specific antigen
qPCR: quantitative polymerase chain reaction
RIP: RNA immunoprecipitation
RNA-FISH: RNA fluorescence in situ hybridization
TCGA: The Cancer Genome Atlas
WCL: Whole-cell lysate
