# Supplementary material for: A novel AR translational regulator lncRNA LBCS inhibits castration resistance of prostate cancer
Source: Mol Cancer. 2019 Jun 20;18:109. doi: 10.1186/s12943-019-1037-8 (PMC6585145; doi:10.1186/s12943-019-1037-8)
Supplement: Supplementary file 1 — Table S1. The sequences of siRNAs and shRNAs. (DOCX 13 kb) [file 12943_2019_1037_MOESM1_ESM.docx]

**Table S1.** The sequences of siRNAs and shRNAs are listed as follows.

| Name | Sequence 5’-3’ |
| --- | --- |
| Sh-Ctrl | CAACAAGATGAAGAGCACCAA |
| Sh-LBCS-1 | AAGCTGCTCATTTGTCTGATC |
| Sh-LBCS-2 | CTGCTCATTTGTCTGATCTAT |
| Si-Ctrl | UUCUCCGAACGUGUCACGUTT |
| Si-hnRNPK | GGGUUGUAGAGUGCAUAAATT |
