# Supplementary material for: A novel AR translational regulator lncRNA LBCS inhibits castration resistance of prostate cancer
Source: Mol Cancer. 2019 Jun 20;18:109. doi: 10.1186/s12943-019-1037-8 (PMC6585145; doi:10.1186/s12943-019-1037-8)
Supplement: Supplementary file 2 — Table S2. The primers used in real time qPCR. (DOCX 13 kb) [file 12943_2019_1037_MOESM2_ESM.docx]

**Table S2.** The primers used in real time qPCR are listed as follows.

| Primer Name | Sequence 5’-3’ |
| --- | --- |
| LBCS Forward | CTCACTCGCTTGCTTGCGCTG |
| LBCS Reverse | TATCCACCAGGAGGGTTTTGC |
| AR Forward | TGAGCAGAGTGCCCTATCCCA |
| AR Reverse | CTGGGGTGGAAAGTAATAGTC |
| PSA Forward | GTATCACGTCATGGGGCAGTG |
| PSA Reverse | GTTGGCCACGATGGTGTCCTT |
| TMPRSS2 Forward | GTGAAACCAGTGTGTCTGCCC |
| TMPRSS2 Reverse | CACCTTGGCAGCGTTCAGCA |
| OPRK1 Forward | AACTCGCTGGTCATGTTCGT |
| OPRK1 Reverse | CTCTGAAAGGGCATGGTTGT |
| U6 Forward | CTCGCTTCGGCAGCACATATAC |
| U6 Reverse | AACGCTTCACGAATTTGCGTGTC |
| Lnc-p21 Forward | GGGTGGCTCACTCTTCTGGC |
| Lnc-p21 Reverse | TGGCCTTGCCCGGGCTTGTC |
| GPADH Forward | CAAGGCTGAGAACGGGAAG |
| GPADH Reverse | TGAAGACGCCAGTGGACTC |
