# Supplementary material for: A novel AR translational regulator lncRNA LBCS inhibits castration resistance of prostate cancer
Source: Mol Cancer. 2019 Jun 20;18:109. doi: 10.1186/s12943-019-1037-8 (PMC6585145; doi:10.1186/s12943-019-1037-8)
Supplement: Supplementary file 3 — Table S3. The probes used in this article. (DOCX 14 kb) [file 12943_2019_1037_MOESM3_ESM.docx]

**Table S3.** The probes used in this article are listed as follows.

| Probe Name | Sequence 5’-3’ | Label | Application |
| --- | --- | --- | --- |
| LBCS | TAGGAAGTGAGCTGTAGACATA | 5’-and 3’-DIG | ISH |
| Scramble | GTGTAACACGTCTATACGCCCA | 5’-and 3’-DIG | ISH |
| beta-actin | CTCATTGTAGAAGGTGTGGTGCCA | 5’-and 3’-DIG | ISH |
| U6 | CACGAATTTGCGTGTCATCCTT | 5’-and 3’-DIG | ISH |
| LBCS | ATTTATGCTATCCACCAGGA | 5’-and 3’-CY3 | FISH |
| U6 | CACGAATTTGCGTGTCATCCTT | 5’-and 3’-CY3 | FISH |
| LBCS-even-1 | AGCGCAAGCAAGCGAGTGAG | 3’-Biotin | RNA purification |
| LBCS-even-2 | ATTTATGCTATCCACCAGGA | 3’-Biotin | RNA purification |
| LBCS-even-3 | AGGGCTTGTTTTTACTGGAA | 3’-Biotin | RNA purification |
| LBCS-even-4 | AGGTTTACGAAAGAGTCCCT | 3’-Biotin | RNA purification |
| LBCS-even-5 | CTGTTTAGGAAGTGAGCTGT | 3’-Biotin | RNA purification |
| LBCS-odd-6 | CCCGTGGGAAGTAAGTGAAA | 3’-Biotin | RNA purification |
| LBCS-odd-1 | GTCAGTTGTGGACTTTCTTC | 3’-Biotin | RNA purification |
| LBCS-odd-2 | CATAGATCAGACAAATGAGC | 3’-Biotin | RNA purification |
| LBCS-odd-3 | ACAAAGCGAAGAACATGCTG | 3’-Biotin | RNA purification |
| LBCS-odd-4 | CCAACCGAATGCCTTGTGTG | 3’-Biotin | RNA purification |
| LBCS-odd-5 | CTACACATTCTAATGTATTG | 3’-Biotin | RNA purification |
| LBCS-odd-6 | TTTGTGTTGACTACCACCAG | 3’-Biotin | RNA purification |
