# Supplementary material for: A novel AR translational regulator lncRNA LBCS inhibits castration resistance of prostate cancer
Source: Mol Cancer. 2019 Jun 20;18:109. doi: 10.1186/s12943-019-1037-8 (PMC6585145; doi:10.1186/s12943-019-1037-8)
Supplement: Supplementary file 4 — Table S4. The primers used in ChIP-real time qPCR. (DOCX 13 kb) [file 12943_2019_1037_MOESM4_ESM.docx]

**Table S4.** The primers used in ChIP-real time qPCR are listed as follows.

| Primer Name | Sequence 5’-3’ |
| --- | --- |
| PSA-P Forward | GCCTGGATCTGAGAGAGATATCATC |
| PSA-P Reverse | ACACCTTTTTTTTTCTGGATTGTTG |
| TMPRSS2-P Forward | GGTAAACTCTCCCTGCCACA |
| TMPRSS2-P Reverse | TACTCCAGGAAGTGGGGATG |
| OPRK1-P Forward | CTGCTCCTGGCATTATCCTC |
| OPRK1-P Reverse | TGTGGCTCTCAGCAGGAAGT |
| Negative control F | GTAATCAGGAAACTGCATAC |
| Negative control R | CTCAAGACTCAATAGTGATC |
