# Supplementary material for: A novel AR translational regulator lncRNA LBCS inhibits castration resistance of prostate cancer
Source: Mol Cancer. 2019 Jun 20;18:109. doi: 10.1186/s12943-019-1037-8 (PMC6585145; doi:10.1186/s12943-019-1037-8)
Supplement: Supplementary file 5 — Table S5. The primers used for RNA isolation by RNA purification-real time qPCR. (DOCX 13 kb) [file 12943_2019_1037_MOESM5_ESM.docx]

**Table S5.** The primers used for RNA isolation by RNA purification-real time qPCR are listed as follows.

| Primer Name | Sequence 5’-3’ |
| --- | --- |
| AR1-chirp-F | CCCGAGTTTGCAGAGAGGTAAC |
| AR1-chirp-R | CTCCGCGTGCAGCCTAACCAG |
| AR2-chirp-F | CTGTTTTCCCCCACTCTCTCTC |
| AR2-chirp-R | TGGCTACTGAAGACCTGACTGC |
| AR3-chirp-F | CCCTTTCCTCTTCGGTGAAGT |
| AR3-chirp-R | AGGACAAAGGCAGCCGTCAGT |
| GAPDH-F | CAAGGCTGAGAACGGGAAG |
| GAPDH- R | TGAAGACGCCAGTGGACTC |
