# Supplementary material for: A novel AR translational regulator lncRNA LBCS inhibits castration resistance of prostate cancer
Source: Mol Cancer. 2019 Jun 20;18:109. doi: 10.1186/s12943-019-1037-8 (PMC6585145; doi:10.1186/s12943-019-1037-8)
Supplement: Supplementary file 6 — Table S6. Actual p-values of all figures. (DOCX 16 kb) [file 12943_2019_1037_MOESM6_ESM.docx]

**Table S6.** Actual p-values of all figures are listed as follows.

| Figure | Column 1 | Column 2 | Column 3 | Column 4 | Column 5 | Column 6 |
| --- | --- | --- | --- | --- | --- | --- |
| 1A | 0.020/  0.024 | 0.18/0.024 | 0.006/  0.054 | 0.011/  0.013 | 0.008/  0.016 |  |
| 1B | 0.49/0.82 | 0.88/0.74 | 0.18/0.11 | 0.040/  0.033 | 0.0050/  0.0060 | 0.0040/  0.0036 |
| 1C | 0.0047 | 0.0042 | 0.0033 |  |  |  |
| 2A | <0.0001 | <0.0001 |  |  |  |  |
| 2B | 0.0047 | 0.0033 |  |  |  |  |
| 2C | 0.7 | 0.32 | 0.056 | 0.011 | 0.0032 | 0.0035 |
| 2D | 0.058 | 0.66 | 0.24 | 0.060 | 0.0043 | 0.011 |
| 2E | 0.18 | 0.34 | 0.29 | 0.060 | 0.025 | 0.0030 |
| 2H | 0.0081 | 0.012 | 0.02 | 0.0090 |  |  |
| 2I | 0.85 | 0.18 | 0.0090 | 0.0090 | 0.37 |  |
| 2J | 0.87 | 0.036 | 0.0079 | 0.034 | 0.020 |  |
| 2K | 0.90 | 0.10 | 0.0048 | 0.0014 | 0.061 |  |
| 2L | 0.010 | 0.00075 | 0.031 | 0.037 |  |  |
| 2M | 0.62 | 0.72 | 0.0049 | 0.0039 | 0.81 | 0.010 |
| 2N | 0.34 | 1.00 | 0.011 | 0.0053 | 0.062 | 0.015 |
| 2O | 0.84 | 0.73 | 0.0029 | 0.067 | <0.0001 | 0.011 |
| 3A | <0.0001 | 0.76 | 0.0090 | 0.030 | 0.046 |  |
|  | <0.0001 | 0.67 | 0.0087 | 0.039 | 0.019 |  |
|  | 0.0064/  0.0080 | 0.48/  0.88 | 0.0023/  0.0031 | 0.0033/  0.0049 | 0.012/  0.034 |  |
| 3C | 0.017 | 0.026 | 0.047 | 0.029 |  |  |
| 3I | 0.19/0.061 | 0.070/  0.082 | 0.083/0.33 |  |  |  |
|  | 0.40/0.74 | 0.011/  0.011 | 0.010/  0.011 |  |  |  |
|  | 0.59/0.14 | 0.026/  0.010 | 0.011/  0.016 |  |  |  |
|  | 0.53/0.22 | 0.024/  0.010 | 0.019/  0.014 |  |  |  |
| 4C | 0.0075 | 0.78 | 0.0049 | 0.43 | 0.67 |  |
| 4D | 0.44 | 0.72 | 0.61 |  |  |  |
| 4J | <0.0001/  <0.0001 | <0.0001/  <0.0001 | 0.089/0.21 | 0.38/0.052 | 0.29/0.55 |  |
| 4L | 0.0049 | 0.43 |  |  |  |  |
| 5C | 0.77 | 0.0040 | 0.0041 | 0.00076 |  |  |
|  | 0.32 | 0.00039 | 0.00068 | 0.00081 |  |  |
| 5D | 0.00041 | 0.00053 | 0.0073 | 0.00071 | Column  1 vs. 3  0.00071 | Column  2 vs. 4  0.0041 |
|  | 0.00067 | 0.00098 | 0.015 | 0.025 | Column  1 vs. 3  0.015 | Column  2 vs. 4  0.017 |
| S2A | 0.026 | 0.0085 | 0.043 | 0.042 | 0.0057 | 0.087 |
| S2B | 0.047/  0.018 | 0.015/  0.0034 | 0.38/0.34 |  |  |  |
| S2C | 0.59 | 0.0022 | 0.75 | 0.017 |  |  |
| S2D | 0.82/0.25 | 0.0034/  0.0019 |  |  |  |  |
