# Supplementary material for: A novel AR translational regulator lncRNA LBCS inhibits castration resistance of prostate cancer
Source: Mol Cancer. 2019 Jun 20;18:109. doi: 10.1186/s12943-019-1037-8 (PMC6585145; doi:10.1186/s12943-019-1037-8)
Supplement: Supplementary file 7 — Supplemental Material and Method. (DOCX 17 kb) [file 12943_2019_1037_MOESM7_ESM.docx]

**Supplemental Material and Method**

**Lentivirus transduction**

To establish stable overexpression and knockdown cell lines, full length lnc-LBCS or shRNA sequences that specifically target lnc-LBCS were cloned into vectors of pLenti-EF1a-Puro-CMV-MCS or pLKO.1-Puro. Bidirectional sequencing was performed to verify the correct sequences. The sequences of all shRNAs are listed in Additional file 1: Table S1. Lentivirus production and infection were conducted as described previously.

***In situ* hybridization (ISH)**

After dewaxing and rehydration, the samples were digested with 20 μg/ml proteinase K (Roche), fixed in 4% paraformaldehyde, hybridized with a double (5’and 3’)-digoxin-labeled locked nucleic acid (LNA)-modified Lnc-LBCS probe (Exiqon) at 42 °C overnight, and subsequently incubated overnight at 4 °C with an anti-digoxin monoclonal antibody conjugated to alkaline phosphatase (Roche). After staining with nitroblue tetrazolium/5-bromo-4-chloro-3-indolylphosphate (Roche), the sections were mounted and observed. A double (5’ and 3’)-DIG labeled scrambled probe was used as the negative control, and double (5’ and 3’)-DIG-labeled probes against U6 snRNA (nuclear staining) were used as technical positive controls. The sequences of all the probes used in ISH are shown in Additional file 3: Table S3.

**Cell cycle analysis by flow cytometry**

For the cell cycle analysis, cells were harvested and fixed in 70% ice-cold ethanol and followed by RNase A treatment, and stained with 50 μg/mL of propidium iodide. All analyses were conducted on a FACSCaliber BD flow cytometer (Franklin Lakes, New Jersey, USA). The data were collected and processed using the BD FACSuite analysis software.

**Detection of caspase-3/7 activity**

Cells were treated with either IC_50_ bicalutamide or control for 48 hours. Then the enzymatic activity of caspase-3/7 was measured using the Caspase-Glo 3/7 Assay kit (Promega, Shanghai, China) according to manufacturer’s instruction.

**Chromatin immunoprecipitation (ChIP) assay and RNA isolation by RNA purification**

ChIP was conducted according to manufacturer's instructions. Stable transfected cells were treated with 1% formaldehyde for 10 min, lysed with SDS lysis buffer, followed by ultrasonication, and then incubated with appropriate antibodies (anti-hnRNPK or RNA polymerase-II). After washing by low salt, high salt, and LiCl buffer, the elution buffer was used to harvest the chromatin fragments. Finally, de-crosslinking was performed and enrichment was examined using qPCR. Primers for ChIP-qPCR are listed in Additional file 4: Table S4.

The RNA isolation by RNA purification was conducted according to the manufacturer’s instructions. Briefly, 3’-end Biotin-TEG modified-DNA probes against lnc-LBCS and TERC were synthesized by Sangon. The sequences of the probes are available in Supplementary Table. LNCaP cells (2×10^7^) were cross-linked for each hybrid reaction. The cell lysate was then sonicated to shear the chromatin to 100~200 bp fragments. The sonicated cell lysates were hybridized with a mixture of biotinylated DNA probes for 4 h at 37 °C. The binding complexes were then recovered using streptavidin-conjugated magnet beads. Finally, the RNA was eluted and purified from the beads for qPCR analyses. The LacZ probe set was provided along with the kit to serve as a negative control probe. The primers used for qPCR are listed in Additional file 5: Table S5.

**Co-IP**

pCMV-FLAG-hAR was a gift from Elizabeth Wilson (Addgeneplasmid#89080 ; http://n2t.net/addgene:89080 ; RRID:Addgene_89080). HA-Ubiquitin was a gift from Edward Yeh (Addgene plasmid#18712; http://n2t.net/addgene:18712; RRID:Addgene_18712). Cells with co-expression of AR (tagged with Flag) and HA-Ub were lysed in IP lysis buffer (50mM Tris-Cl pH7.4, 150mM NaCl, 1mM EDTA, 1% Triton X-100, Roche protease inhibitor cocktail). An aliquot of the cell lysate was kept as input for Western blot analysis. Cell lysate was first pre-cleared with protein A/G-magnetic beads (Thermo Scientific) at 4°C for 2 h. Then the pre-cleared lysate was incubated with rabbit anti-Flag (1:2000, Abcam) or mouse IgG overnight on a rocker platform. The next day, protein A/G- magnetic beads were added to the mixture and incubated at 4°C for 2h. Then beads were pelleted and washed four times with IP lysis buffer before boiling in SDS sample buffer. Western blotting analysis were performed using mouse anti-Ub (1:2000, Abcam) according to protocol as described previously.

**Fluorescence resonance energy transfer (FRET)**

FRET was performed as previously described. RNA-RNA comples formation was assayed in binding buffer (20 mM HEPES pH 7.5, 50 mM Na-acetate, 10 mM MgCl2). AR mRNA (500 nM) labeled with 6-carboxyfluorescein (FAM) and 500 nM LBCS RNA labeled with 5-carboxytetramethylrhodamine (TAMRA) were mixed, annealed by heating at 55 °C for 10 min and equilibrated at 37 °C. Fluorescence was measured using a FluoroMax-4 Spectrofluorometer (HORIBA). Excitation was at 470 nm, and emission was determined between 490 and 640 nm. To calculate the efficiency of FRET, we used the fluorescence of the acceptor group (TAMRA), which was obtained by averaging the fluorescence emission spectra between 576 and 584 nm. The oligos for FRET are listed in Additional file 14: Table S10.

**Luciferase assay and site directed mutagenesis**

The indicated regions of the AR UTR were cloned into the psiCHECK2 luciferase reporter plasmid and transfected into the 293T cells. The pcDNA3.1-lnc-LBCS or control vector were co-transfected into cells, respectively. The luciferase activity was detected according to the manual of the Dual-Luciferase Reporter Assay system (Promega) at 48h after transfection. Firefly luciferase activity was normalized against Renilla luciferase activity. Site directed mutagenesis was conducted using Phusion Site-Directed Mutagenesis Kit (ThermoFisher), the primers were listed in Additional file 8: Table S7.
