# Supplementary material for: A novel AR translational regulator lncRNA LBCS inhibits castration resistance of prostate cancer
Source: Mol Cancer. 2019 Jun 20;18:109. doi: 10.1186/s12943-019-1037-8 (PMC6585145; doi:10.1186/s12943-019-1037-8)
Supplement: Supplementary file 8 — Table S7. The primers used in gene clone (DOCX 14 kb) [file 12943_2019_1037_MOESM8_ESM.docx]

**Table S7.** The primers used in gene clone are listed as follows.

| Primer Name | Sequence 5’-3’ |
| --- | --- |
| AR5’-UTR Forward | AATCTCGAGCGAGATCCCGGGGAGCCAGCTTGCT |
| AR5’-UTR Reverse | GTGAAGCTTCCTTGAGCTTGGCTGAATCTTCCAC |
| AR5’-UTR(1-300) Forward | AATCTCGAGCGAGATCCCGGGGAGCCAGCTTGC |
| AR5’-UTR(1-300) Reverse | GTGAAGCTTGACAAAGGCAGCCGTCAGTCCTAC |
| AR5’-UTR(282-620) Forward | AATCTCGAGACTGACGGCTGCCTTTGTCCTCCT |
| AR5’-UTR(282-620) Reverse | GTGAAGCTTAAGGCAGGAGGAGGTGGAGAGAGA |
| AR5’-UTR(620-871) Forward | ACTCTCGAGTCTCTCTCCACCTCCTCCTGCCTT |
| AR5’-UTR(620-871) Reverse | GTGAAGCTTCCTGCTAGGCTCACAGTCTGTCTC |
| AR5’-UTR(864-1115) Forward | GCCCTCGAGGTATTAAGAGACAGACTGTGAGCC |
| AR5’-UTR(864-1115) Reverse | GTGAAGCTTCCTTGAGCTTGGCTGAATCTTCCA |
| AR5’-UTR(507-627) Forward | CATCTCGAGCACATTGCAAAGAAGGCTCTTAGGAG |
| AR5’-UTR(507-627) Reverse | GTGAAGCTTAGAGAGAGTGGGGGAAAACAGAGGGT |
| AR5’-UTR(507-627)mut Forward | GACCGTCATTAGAACTCCTGCCACGACCCG |
| AR5’-UTR(507-627)mut Forward | CCCAGTCGCCTGGCTCCTAAGAGCCTTCTTT |
| AR3’-UTR (1-2025)Forward | GATCTCGAGAGCATTGGAAACCCTATTTCCCCACC |
| AR3’-UTR (1-2025) Reverse | GGCAAGCTTAAAGACTATATAAAACCAGTGTCCCA |
| AR3’-UTR(2026-3980)Forward | TAGCTCGAGTGGCACACCTGTGTTCTGTTGACTTC |
| AR3’-UTR(2026-3980)Reverse | GGCAAGCTTAAACATGAAAACCACTCCCTTGGCCC |
| AR3’-UTR(3950-6742)Forward | TAGCTCGAGCACTGGGCCAAGGGAGTGGTTTTCAT |
| AR3’-UTR(3950-6742)Reverse | GCCAAGCTTAACAATGATAAGAGGACATTAGCAGC |
