# Supplementary figures and images for: A novel AR translational regulator lncRNA LBCS inhibits castration resistance of prostate cancer
Source: Mol Cancer. 2019 Jun 20;18:109. doi: 10.1186/s12943-019-1037-8 (PMC6585145; doi:10.1186/s12943-019-1037-8)

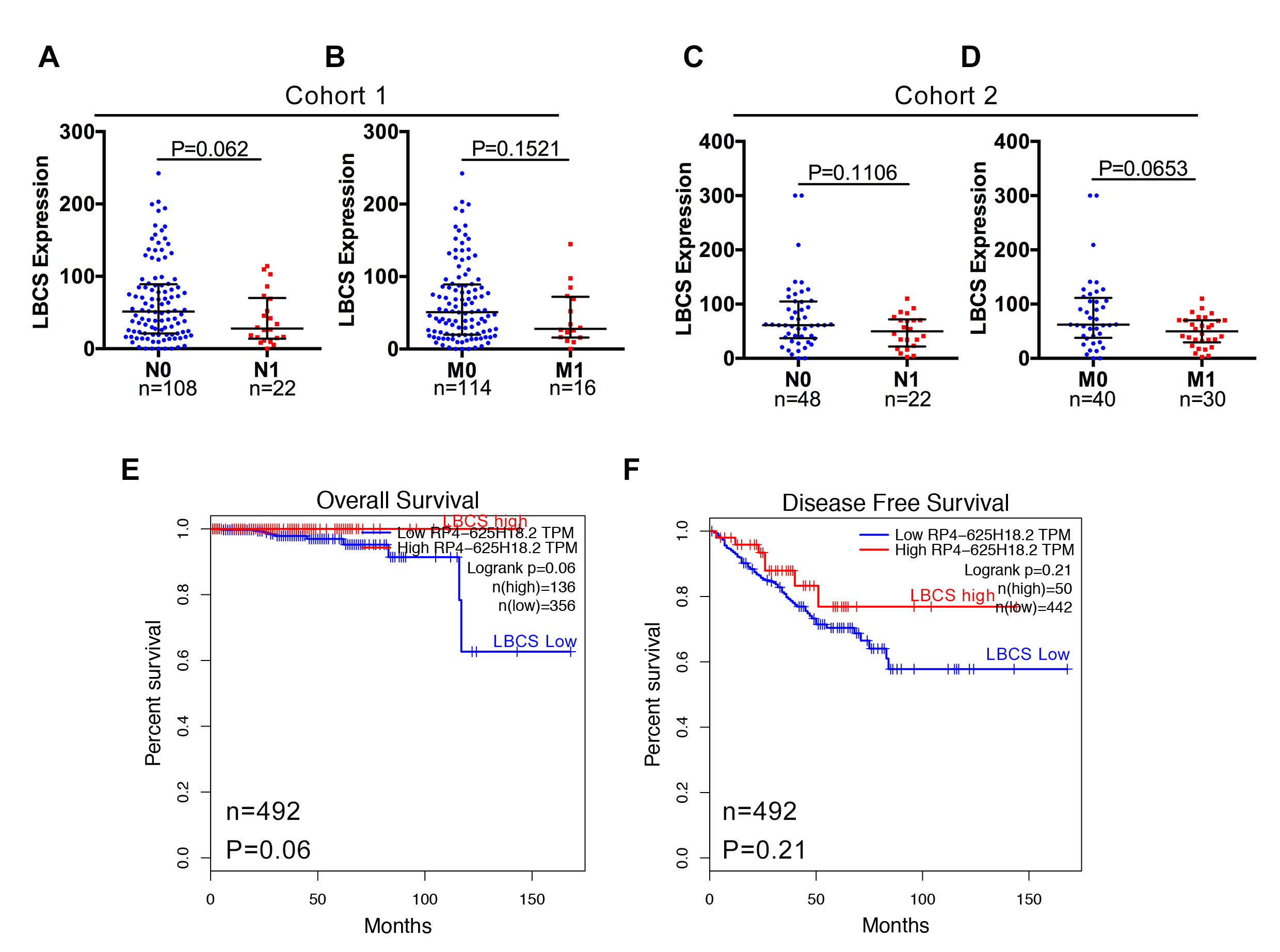

Supplement: Supplementary file 9 — Figure S1. The clinical significance of lnc-LBCS in prostate cancer. (A-B) Lnc-LBCS was detected between N0 and N1, M0 and M1 groups from cohort 1 by ISH. ISH of lnc-LBCS expression was quantified by the expression score (0–300). Patients with unavailable information was excluded for analysis. The whiskers indicate median ± interquartile in the plots. (C-D) Lnc-LBCS was detected between N0 and N1, M0 and M1 groups from cohort 2 by ISH. ISH of lnc-LBCS expression was quantified by the expression score (0–300). Patients with unavailable information was excluded for analysis. The whiskers indicate median ± interquartile in the plots. (E-F) The overall survival and disease-free survival rates of the 492 PCa patients from TCGA were analyzed by GEPIA. *p < 0.05, **p < 0.01. (JPG 1018 kb) [file 12943_2019_1037_MOESM9_ESM.jpg]

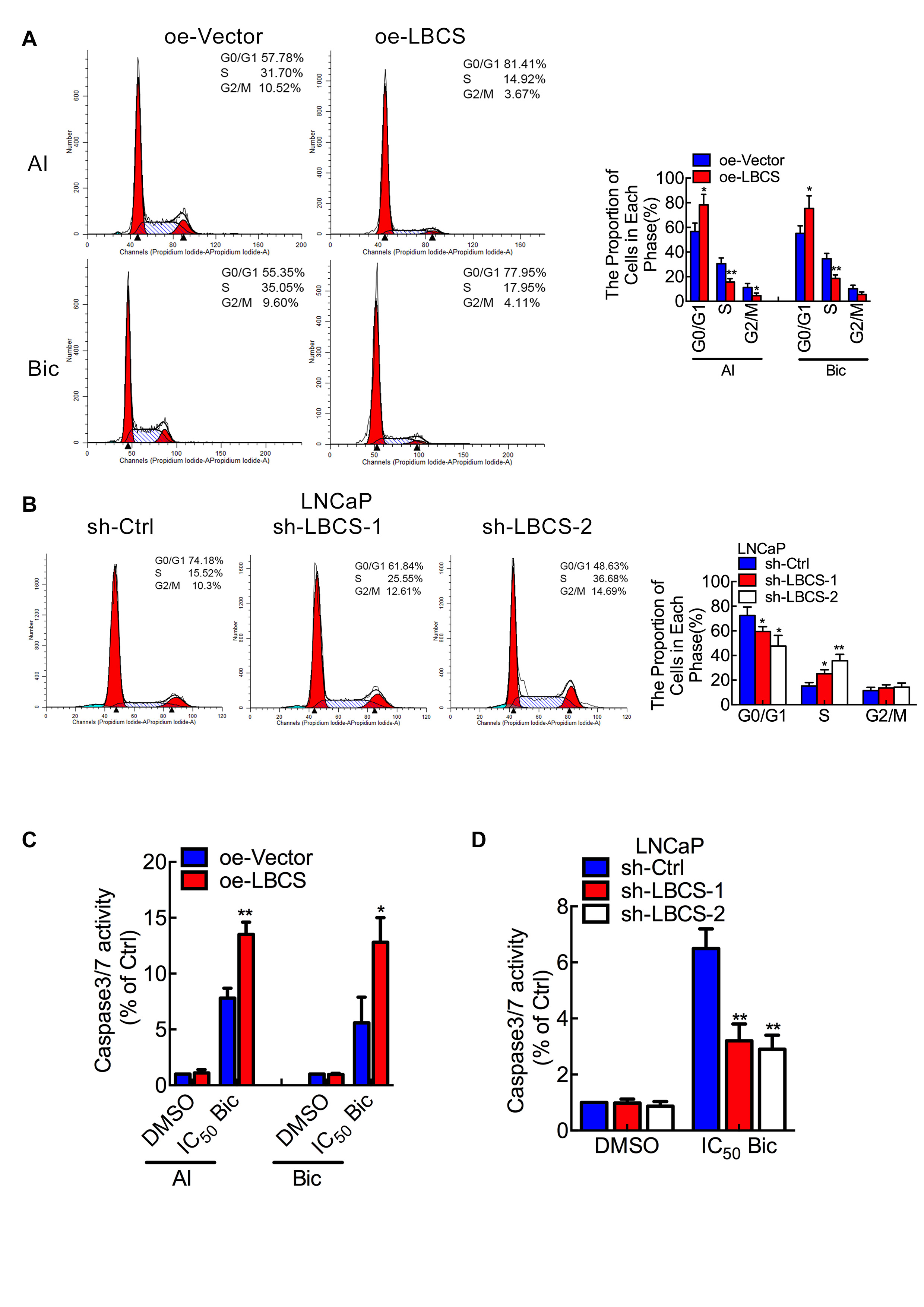

Supplement: Supplementary file 12 — Figure S2. LBCS restores the castration sensitivity of CRPC cells. (A-B) LBCS was overexpressed in LNCaP-AI and LNCaP-Bic cells and knocked down in LNCaP cells, then cell cycles were analyzed by flow cytometry. (C-D) The caspase 3/7 activity was measured in lnc-LBCS overexpressed LNCaP-AI and LNCaP-Bic cells, and lnc-LBCS knockdown LNCaP cells treated with bicalutamide. *p < 0.05, **p < 0.01. (JPG 922 kb) [file 12943_2019_1037_MOESM12_ESM.jpg]

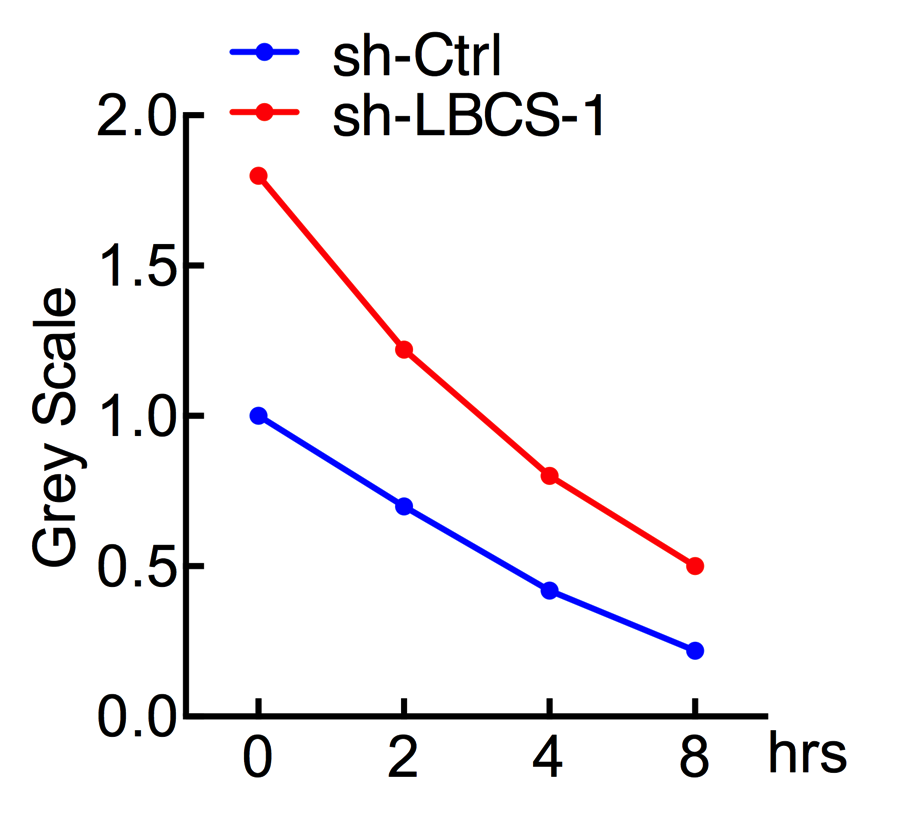

Supplement: Supplementary file 13 — Figure S3. The illustration of the change of AR grey scale after treated with cycloheximide for different hours in either LBCS knockdown or control group. (TIFF 93 kb) [file 12943_2019_1037_MOESM13_ESM.tiff]
