# Supplementary material for: A novel AR translational regulator lncRNA LBCS inhibits castration resistance of prostate cancer
Source: Mol Cancer. 2019 Jun 20;18:109. doi: 10.1186/s12943-019-1037-8 (PMC6585145; doi:10.1186/s12943-019-1037-8)
Supplement: Supplementary file 10 — Table S8. Association between lnc-LBCS expression and clinicopathological features of prostate cancer from Cohort 2. (DOCX 15 kb) [file 12943_2019_1037_MOESM10_ESM.docx]

**Table S8**

Association between lnc-LBCS expression and clinicopathological features of prostate cancer from Cohort 2

| **Characteristics** | **Cases(%)** | | | ***χ^2^*** | ***P*-value** |  |
| --- | --- | --- | --- | --- | --- | --- |
| **Total Cohort 2 Patients(N)** | 70 | | |  |  |  |
| **LBCS expression** | Low | High | |  | |  |
| **Age(Year)** |  | |  |  |  | |
| ≤70 | 24(34) | | 22(31) | 0.615 | 0.802 | |
| >70 | 11(16) | | 13(19) |  |  |  |
| **Gleason Score** |  | |  |  |  |  |
| 6-7 | 12(17) | | 22(31) | 5.719 | **0.017*** | |
| 8-10 | 23(33) | | 13(19) |  |  |  |
| **Tumor stage** |  | |  |  |  | |
| T2 | 16(23) | | 25(36) | 4.769 | **0.029*** | |
| T3-4 | 19(27) | | 10(14) |  |  |  |
| **Lymphnodes status N** |  | |  |  |  | |
| Negative | 22(32) | | 26(54) | 1.061 | 0.303 | |
| Positive | 13(3) | | 9(11) |  |  |  |
| **Distant Metastasis M** |  | |  |  |  | |
| M0 | 17(24) | | 23(33) | 2.100 | 0.147 | |
| M1 | 17(25) | | 13(18) |  |  |  |

**P*< 0.05 is considered significant.

Median H-Score of LBCS was used as cut-off value for analysis
