# Supplementary material for: A novel AR translational regulator lncRNA LBCS inhibits castration resistance of prostate cancer
Source: Mol Cancer. 2019 Jun 20;18:109. doi: 10.1186/s12943-019-1037-8 (PMC6585145; doi:10.1186/s12943-019-1037-8)
Supplement: Supplementary file 11 — Table S9. Univariate and multivariate analysis of factors associated with progression-free survival in prostate cancer Cohort 1. (DOCX 15 kb) [file 12943_2019_1037_MOESM11_ESM.docx]

**Table S9**

Univariate and multivariate analysis of factors associated with progression-free survival in prostate cancer cohort 1.

|  | | Univariate | | |  | Multivariate | | |
| --- | --- | --- | --- | --- | --- | --- | --- | --- |
| Variable | | HR | 95% CI | *p* |  | HR | 95% CI | *p* |
| Age, years (>70/≤70) | | 1.158 | 0.509–2.631 | 0.727 |  |  |  | NA |
| Gleason Score (8-10/6-7) | | 1.967 | 0.860-4.495 | 0.109 |  |  |  | NA |
| Tumor stage (T3-4/T1-2) | | 2.061 | 0.906-4.690 | 0.085 |  |  |  | NA |
| Nodal metastasis (N1/N0) | | 2.087 | 0.757-5.757 | 0.155 |  |  |  | NA |
| Distant metastasis(M1/M0) | | 2.558 | 0.843-7.766 | 0.097 |  |  |  | NA |
| LBCS (high/low) | | 0.397 | 0.168-0.938 | **0.035** |  |  |  | NA |
|  | Univariate and multivariate analysis. Cox proportional hazards regression model. Variables associated with survival by univariate analyses were adopted as covariates in multivariate analyses. Significant P-values are shown in bold font. HR > 1, risk for death increased; HR < 1, risk for death reduced. Median H-Score of LBCS was used as cut-off value for analysis. | | | | | | | |
