# Supplementary material for: A novel AR translational regulator lncRNA LBCS inhibits castration resistance of prostate cancer
Source: Mol Cancer. 2019 Jun 20;18:109. doi: 10.1186/s12943-019-1037-8 (PMC6585145; doi:10.1186/s12943-019-1037-8)
Supplement: Supplementary file 14 — Table S10. The predicted LBCS-AR mRNA interacting sequences and oligos used for FRET. (DOCX 14 kb) [file 12943_2019_1037_MOESM14_ESM.docx]

**Table S10.** The predicted LBCS-AR mRNA interacting sequences and oligos used for FRET are listed as follows.

| Oligo Name | Sequence 5’-3’ | Label |
| --- | --- | --- |
| LBCS-1 | AAATCTGCTTTGTTGAAGCTGCTCATTTGTC | 5’- TAMRA |
| AR-1 | GTGTAACACGTCTATACGCCCA | 5’- FAM |
| LBCS-2 | CTCATTGTAGAAGGTGTGGTGCCA | 5’- TAMRA |
| AR-2 | CACGAATTTGCGTGTCATCCTT | 5’- FAM |
| LBCS-3 | ATTTATGCTATCCACCAGGA | 5’- TAMRA |
| AR-3 | CACGAATTTGCGTGTCATCCTT | 5’- FAM |
| LBCS-mut-1 | AGCGCAAGCAAGCGAGTGAG | 5’- TAMRA |
